# Biomarker-driven neoadjuvant immunotherapy in triple-negative breast cancer: emerging therapeutic targets FOXP3 and WT1

**DOI:** 10.3389/fimmu.2026.1851289

**Published:** 2026-08-10

**Authors:** Ziwei Zhang, Renjie Zhao, Yuan Hu, Qiyu Xie, Yan Yu, Kefan Li, Zhenyu Ding, Manni Wang

**Affiliations:** 1 State Key Laboratory of Biotherapy and Cancer Center, Department of Biotherapy, West China Hospital, Sichuan University, Chengdu, China; 2 Department of Pediatric Urinary Disease Center Nursing, West China Second University Hospital, Sichuan University, Chengdu, China; 3 West China School of Nursing, Sichuan University, Key Laboratory of Birth Defects and Related Diseases of Women and Children (Sichuan University), Ministry of Education, Chengdu, Sichuan, China; 4 Department of Orthopedic Surgery and Orthopedic Research Institution, West China Hospital, Sichuan University, Chengdu, China

**Keywords:** triple-negative breast cancer, neoadjuvant immunotherapy, biomarker, PD-1/PD-L1, FOXP3, WT1, clinical trials

## Abstract

Despite advances in first-line therapy, a subset of breast cancer patients—particularly those with triple-negative breast cancer (TNBC)—continue to experience unfavorable prognosis. Recently, the addition of neoadjuvant immunotherapy has been intensively studied and widely adopted to improve clinical outcomes of patients with breast cancer. Based on encouraging results from large-scale clinical trials such as KEYNOTE-522 and IMpassion031, the range of drugs evaluated has expanded from atezolizumab to other programmed cell death protein 1/programmed cell death-ligand 1(PD-1/PD-L1) inhibitors, such as pembrolizumab and avelumab. Furthermore, the pursuit of targeted therapies has extended to other biomarkers, including Forkhead Box Protein P3 (FOXP3) and Wilms’ Tumor gene 1 (WT1). Despite these advancements, the heterogeneity in trial design, participant demographics, endpoints, and other confounding variables has resulted in divergent conclusions across clinical studies. In this review, we delineate the principal neoadjuvant immunotherapy biomarkers implicated in breast cancer and elucidate their action mechanisms. We also summarize the ongoing and reported clinical studies, aiming to offer a coherent perspective on the evolving role of neoadjuvant immunotherapy in breast cancer management.

## 1 Introduction

Breast cancer has emerged as one of the most prevalent and fatal neoplastic diseases, exerting a significant health burden across the globe (1, 2). Despite efforts of laboratory, epidemiological, and clinical research, the incidence of breast cancer remains on an upward trajectory, with a prevalence of 12.5% in developed countries (3). In 2020, breast cancer has become the leading cause of cancer mortality in women globally, with an estimated 2.3 million new cases (4, 5). In 2022, lung and breast cancers were the most commonly diagnosed cancers worldwide (6).

The selection of breast cancer treatment depends on the stage of the disease at diagnosis. Early breast cancer is potentially curable with multimodal treatment incorporating surgery, with radiotherapy and systemic therapy as indicated (7). Within the spectrum of surgical approaches, both adjuvant and neoadjuvant therapies have been extensively integrated to enhance treatment efficacy and patient outcomes (8–11). Specifically, for the majority of TNBC and early-stage human epidermal growth factor receptor 2-positive (HER2+) breast cancers, a paradigm shift has occurred, with neoadjuvant therapy followed by risk-adapted adjuvant strategies now constituting the standard of care (12). Neoadjuvant therapy is used prior to surgical intervention on operable tumors, aiming to achieve a substantial reduction in tumor or lymph node burden, and facilitate subsequent surgical procedures, which predominantly encompasses neoadjuvant immunotherapy, neoadjuvant chemotherapy (NACT), and combinatorial regimens that integrate both modalities (13–17).

A wealth of research underscores the potential of NACT to influence patient outcomes. In neoadjuvant immunotherapy, studies have shown that the addition of PD-1/PD-L1 blockade to NACT significantly improves pathological complete response (pCR) rates in early TNBC patients, particularly in patients at high risk of relapse (18). For hormone receptor-positive (HR+) and HER2-negative breast cancer, intensive dose-dense regimens such as epirubicin, paclitaxel, and cyclophosphamide (iddEPC) have been shown to significantly benefit patients receiving NACT (19, 20). Moreover, the presence of tumor-infiltrating lymphocytes (TILs) has been identified as a predictive factor of patient prognosis and chemotherapy response (21). Recently, accumulating evidence has proved the efficacy of immune checkpoint inhibitors targeting PD-1/PD-L1, as well as investigational strategies involving FOXP3, WT1, and other biomarkers (22–24). However, several challenges and unanswered questions remain in this field (25, 26). As combinations of NACT and immunotherapy have come to the forefront, heterogeneity in trial design, sample size, endpoints, and other factors has led to sometimes conflicting conclusions. For neoadjuvant immunotherapy, some inhibitors have not been shown to be effective when administered as single-agent therapy. Moreover, the mechanisms underlying the combined action of various therapies remain largely elusive (27–30). The heterogeneity of breast cancer and the complexity of its microenvironment necessitate a personalized approach to therapy. The identification of predictive biomarkers is crucial for patient selection and tailoring of treatments. While PD-L1 expression is the most commonly used biomarker, its predictive value is limited, particularly in metastatic TNBC (31). The development of new biomarkers and their integration into clinical practice are warranted to guide treatment decisions. In this review, we delineate the principal neoadjuvant immunotherapy biomarkers implicated in breast cancer and elucidate their mechanisms of action. We also summarize the ongoing and reported clinical studies, aiming to offer a coherent perspective on the evolving role of neoadjuvant immunotherapy in breast cancer management.

This article was prepared as a narrative review. To improve transparency, we searched PubMed, Web of Science, Embase, Scopus, and ClinicalTrials.gov for English-language articles and registered trials published or updated from January 1997 to June 2026. Search terms included ‘neoadjuvant immunotherapy’, ‘breast cancer’, ‘biomarker’, ‘PD-1’, ‘PD-L1’, ‘FOXP3’, ‘WT1’, ‘immune checkpoint inhibitor’, ‘pathological complete response’, and ‘tumor microenvironment’. We included randomized controlled trials, phase I/II clinical trials, prospective cohort studies, and preclinical studies investigating neoadjuvant immunotherapy in breast cancer. We excluded case reports, conference abstracts without full text, letters, studies focused solely on metastatic breast cancer or HER2-positive breast cancer without neoadjuvant context, and non-English publications. Two authors (ZZ and RZ) independently screened titles and abstracts, then reviewed full texts. Disagreements were resolved through consensus.

## 2 Major biomarkers for neoadjuvant immunotherapy in breast cancer

In neoadjuvant immunotherapy for breast cancer, multiple biomarkers have been identified. We delineate the principal neoadjuvant immunotherapy biomarkers implicated in breast cancer and elucidate their action mechanisms (**Table 1**).

**Table 1:** Major biomarkers for neoadjuvant immunotherapy in breast cancer.

Targets	Model	Result	Reference
CK2	T cells in vitro	PD-1-mediated inhibition of CK2 can result in ubiquitin-dependent degradation and diminished abundance of PTEN protein, which helps the development of resistance to anti-PD-1 therapy.	(32)
PLC-γ1/Ras	MCF-7 and MDA-MB-453 breast cancer cells	PD-1 can be decreased by inhibiting the activation of PLC-γ1 and Ras, which can be blocked by drugs such as 1,25-(OH)2D3, leading to antitumor response.	(33)
ZAP70	Mononuclear cells and T-cell blasts	PD-1 signaling attenuates TCR-induced CD3ζ chain and ZAP70 tyrosine phosphorylation, leading to the suppression of TCR chain.	(34)
TMUB1	Tissue samples from breast cancer patients	TMUB1 enhances PD-L1 N-glycosylation and stability, thereby promoting PD-L1 maturation and tumor immune evasion.	(35)
METTL3	B-NDG and BALB/c mice; breast cancer tissue	METTL3 significantly abolishes m6A modification and reduces the stabilization of PD-L1 mRNA.	(36)
TBM-1	Human/mice cancer cells	TBM-1 disrupts the PD-1/PD-L1 interaction and enhances the cytotoxicity of T cells toward tumor cells through decreasing the abundance of PD-L1.	(37)
CSN5	Immunocompetent BALB/c mice carrying 4T1 cells	CSN5 catalyzes the removal of polyubiquitination from PD-L1 to suppress anti-tumor immune responses during chronic inflammation.	(38)
RBMS3	Nude mice and BALB/c mice+cell lines 293T, MCF-7 and MDA-MB-231	RBMS3 disruption together with AUF is able to decrease the mRNA stability and protein expression of PD-L1, facilitating anti-tumor T-cell immunity.	(39)
VISTA	Tumor mice model	VISTA reduces T-cell proliferation and cytokine production.	(40)
LAIR1	WT mice with immunosuppressive cancer cells implanted	LAIR1 induces T cell exhaustion through SHP-1.	(41)
MTA1	Breast cancer cell lines MDA-MB-231 and MCF-7	FOXP3 inhibits breast cancer metastasis by downregulating the expression of MTA1.	(42)
LAG-3	NOD mouse model	In cooperation with NF-AT, FOXP3 regulates the expression of LAG-3, a CD4-related molecule and mediates tumor immunity.	(43)
CD44	Breast cancer specimens	CD44 induces FOXP3 expression in Tregs and contributes to better prognosis in breast cancer.	(44)
VEGF	MDA-MB-231 tumors transplanted in mice	The inhibitory effects of FOXP3 in the supernatant on tube formation and sprouting in HUVECs could be reversed by adding VEGF *in vitro*.	(45)
LAPTM5	Breast cancer clinical specimens+breast cancer cells+BALB/c female nude mice	LAPTM5, negatively regulated by FOXP3, promotes the malignant phenotypes of breast cancer.	(46)
ZEB2	Breast carcinoma cell lines BT549 and MDA-MB-231	Deregulation of ZEB2 can lead to progression of cancer.	(47)
CKAP2L	Fresh breast cancer and para-carcinoma specimens	CKAP2L can activate AKT/mTOR pathway and promote the progression of breast cancer.	(48)
CXCR4	Breast cancer cell lines MCF-7 and MDA-MB-231	CXCR4 regulates the development of breast cancer by stimulating cell migration toward CXCL12-expressing sites of metastatic spread.	(49)
Runx1	LM3 mouse mammary cancer cell line	Reduced Runx1 transcriptional activity decreases tumor cell migration properties.	(50)
KGF	MCF-7 human breast cancer cell line	KGF-mediated signaling can regulate breast cancer cell proliferation and motility.	(51)
STIM1	Primary Wilms tumor cells	As an essential regulator of Ca2+ entry in non-excitable cells, STIM1 is linked to cancer development.	(52)

### 2.1 PD-1/PD-L1

PD-1 is an immune checkpoint protein expressed on T cells, B cells, NK T cells, monocytes, and dendritic cells (DCs). Upon engagement with its ligand PD-L1—a type I transmembrane glycoprotein of the B7 superfamily expressed on tumor and immune cells—PD-1 triggers intracellular signaling that induces peripheral T-lymphocyte tolerance, attenuates CD4^+^ and CD8^+^ T-cell activity, and facilitates tumor immune evasion (53, 54).

**Figure 1:**
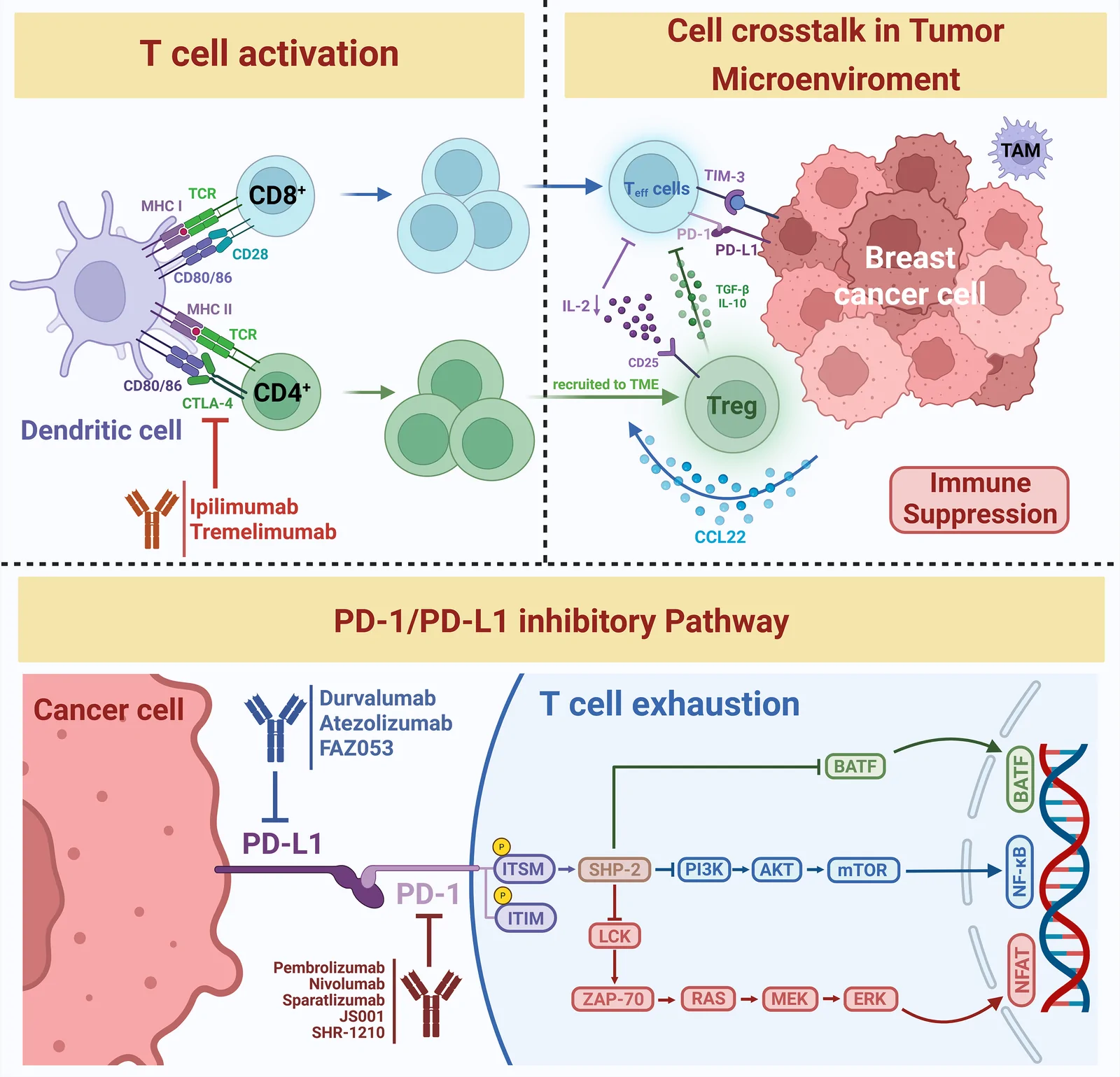
The PD-1/PD-L1 inhibitory pathway. PD-L1 expressed by breast cancer cells engages PD-1 on effector T cells, triggering ITIM/ITSM phosphorylation and SHP-2 recruitment, thereby attenuating proximal TCR signaling. This inhibitory cascade interferes with PI3K–AKT–mTOR and RAS–MEK–ERK signaling and reshapes transcriptional programs involving BATF, NFAT, and NF-κB, ultimately promoting T-cell exhaustion and tumor immune evasion. Therapeutic blockade of PD-1/PD-L1 can restore antitumor T-cell activity. APC, antigen-presenting cell; TCR, T-cell receptor; Teff, effector T cell; TME, tumor microenvironment.

PD-1 can be activated by PD-L1, altering conformation, translocating to dynamic T cell receptor (TCR) microclusters, and accumulating at the signaling central supramolecular activation cluster (c-SMAC) (55, 56). Once the cytoplasmic tail of PD-1 is phosphorylated, the phosphorylated tyrosine motifs such as immunoreceptor tyrosine-based switch motif (ITSM), recruit the phosphatase SH2-containing protein tyrosine phosphatase 2 (SHP-2) near TCR and CD28 signaling (57). **Figure 1** presents the general mechanisms and agents targeting the programmed cell death protein 1 pathway in breast cancer.

In the PD-1/PD-L1 interaction, phosphatidylinositol 3-kinase (PI3K)/protein kinase B (AKT), and RAS-RAF-MAP kinase-ERK kinase (MEK)-extracellular-signal-regulated kinase (ERK) signaling are two major downstream signaling pathways modulated by PD-1/PD-L1 (58). Within the PI3K-AKT cascade, evidence suggests that SHP-2 modulates the ubiquitin-proteasome-dependent degradation of Cyclin D1 via the PI3K/AKT/GSK3β signaling pathway (59, 60). Moreover, activated by the mammalian target of rapamycin (mTOR), AKT phosphorylates target proteins, leading to the stimulation of cell survival, growth, and proliferation (61, 62). The lipid phosphatase PTEN, a key negative regulator of the PI3K signaling, is integral to the modulation of tumor cell migration and angiogenesis (63). PTEN is phosphorylated by casein kinase 2 (CK2) in the Ser380-Thr382-Thr383 cluster within the C-terminal regulatory domain. Therefore, PD-1-mediated inhibition of CK2 can result in ubiquitin-dependent degradation and diminished abundance of PTEN protein, leading to an immunosuppressive tumor microenvironment (TME) which helps the development of resistance to anti-PD-1 therapy (32, 64). In the context of the RAS-RAF-MEK-ERK signaling module, the attenuation of PD-1 expression may be achieved by curbing the activation of phospholipase C gamma 1 and Ras, a process that can be ameliorated by pharmacological agents such as 1,25-dihydroxyvitamin D3, thereby augmenting the antitumor immune response (33). PD-1/PD-L1 can inhibit T cell functions by promoting the expression of transcription factors such as basic leucine zipper transcriptional factor ATF-like (BATF), which represses the expression of downstream effector genes (55). Additionally, PD-1 signaling attenuates TCR-induced CD3ζ chain and Zinc Finger Antiviral Protein 70 (ZAP70) tyrosine phosphorylation, leading to the suppression of TCR chain. In addition, PD-1 signaling attenuates the phosphorylation of protein kinase Cθ (PKCθ) activation loop with a cognate TCR signal, which has been shown as a necessity for IL-2 production (34).

The expression of PD-1 and PD-L1 is intricately regulated by multiple molecules. The PI3K-Akt kinase signaling cascade and the secretion of IFN-γ are instrumental in modulating the expression levels of PD-1/PD-L1 (65). Multiple mechanisms contribute to the upregulation or maturation of PD-1/PD-L1. Scientists identify transmembrane and ubiquitin-like domain-containing protein 1 (TMUB1) as a modulator of PD-L1 post-translational modifications in tumor cells. TMUB1 competes with HECT, UBA and WWE domain-containing protein 1 (HUWE1) to interact with PD-L1. Moreover, by recruiting STT3 oligosaccharyltransferase complex catalytic subunit A (STT3A), TMUB1 enhances PD-L1 N-glycosylation and stability, thereby promoting PD-L1 maturation and tumor immune evasion (35). Additionally, the knockdown of methyltransferase 3, N6-adenosine-methyltransferase complex catalytic subunit (METTL3), significantly reduced N6-methyladenosine (m6A) modification and reduced the stabilization of PD-L1 mRNA, confirming PD-L1 as a downstream target of METTL3-mediated m6A modification in breast cancer cells (36, 66). It has been reported that amplification and mutation of the Janus kinase (JAK) family contribute to PD-L1 upregulation by increasing PD-L1 RNA expression (67). Tri-methylation of histone H3 on lysine 4 (H3K4me3) also boosts PD-L1 expression in cancer cells. For instance, mixed-lineage leukemia 1 (MLL1) can bind directly to the CD274 promoter to catalyze H3K4me3, leading to the upregulation of its protein expression (68, 69). Additionally, targeting MLL1 by inhibitors has been proved to enhance the efficacy of anti-PD-1/PD-L1 therapy.

Conversely, the distribution pattern of PD-L1 can be regulated by the ATPase family AAA domain-containing protein 3-PTEN-induced putative kinase 1 (ATAD3A-PINK1) axis, where PINK1 recruits soluble PD-L1 (sPD-L1) to mitochondria for degradation via a mitophagy pathway (70, 71). Additionally, tubeimoside-1 (TBM-1) disrupts the PD-1/PD-L1 interaction and enhances the cytotoxicity of T cells toward tumor cells through decreasing the abundance of PD-L1 (37). CMTM6 is another factor that impedes the recycling of PD-L1 by obstructing the interaction between STUB1 and PD-L1 (72). Scientists also discovered that the deubiquitinase COP9 signalosome complex subunit 5 (CSN5) catalyzes the removal of polyubiquitination from PD-L1 to suppress anti-tumor immune responses during chronic inflammation (38). It was reported that RNA binding motif single stranded interacting protein 3 (RBMS3) stabilizes CD274 mRNA by interacting with its 3’UTR, which is crucial for PD-L1 upregulation in TNBC. Thus, the disruption of RBMS3 in conjunction with the FDA-approved thioredoxin reductase inhibitor auranofin (AUF) has been demonstrated to diminish PD-L1 mRNA stability and protein expression, thereby reinforcing anti-tumor T-cell immunity (39).

The PD-1/PD-L1 axis is the principal rate-limiting step of the anticancer immune response in breast cancer and other malignancies (73). Tumor-associated macrophages (TAMs) modulate PD-1/PD-L1 expression both directly and indirectly: TGF-β released by TAMs suppresses T-cell activity via Smad2/3 phosphorylation and mitochondrial respiration inhibition (74, 75). Additionally, TAMs can increase the expression of PD-L1 in tumor-infiltrating myeloid cells through the cyclooxygenase-2 (COX-2)/microsomal prostaglandin E synthase-1 (mPGES1)/PGE2 pathway, which leads to the exclusion of CD8+ T cells (76). There are also immune checkpoint ligands on the surface of TAMs that can block anti-PD-1/PD-L1 immune efficacy, such as V-domain Ig-containing suppressor of T-cell activation (VISTA), which reduces T-cell proliferation and cytokine production (40). Therefore, immunotherapy targeting macrophages may be the next step in reversing drug resistance of PD-1/PD-L1 therapy (77–82). Additionally, as a multifunctional growth factor overexpressed in multiple tumors, progranulin (PGRN) is demonstrated to promote M2 macrophages polarization and PD-L1 expression through the signal transducer and activator of transcription 3 (STAT3) signaling pathway. Furthermore, through PD-1/PD-L1 interaction, PGRN can promote the breast tumor immune escape (83). CD18 interaction with collagen can upregulated leukocyte-associated immunoglobulin-like receptor 1 (LAIR1), which induces T cell exhaustion through SHP-1. Therefore, abrogating LAIR1 immunosuppression via LAIR2 overexpression or SHP-1 inhibition to increase the sensitivity of resistant tumors to anti-PD-1/PD-L1 therapy would be a promising therapeutic combination (41). Breast cancer-derived myeloid-derived suppressor cells (MDSCs) could induce PD-1-PD-L1+ regulatory B cells (Bregs) with immunosuppressive functions, which suggests that blocking the PD-1/PD-L1 interaction between MDSCs and B cells could contribute to reversion of an immunosuppressive TME (84).

In summary, the PD-1/PD-L1 axis integrates innate and adaptive immune suppression through a network of signaling, epigenetic, and post-translational mechanisms that are further shaped by the TME. Elucidating this regulatory landscape is essential for optimizing PD-1/PD-L1–targeted therapy in breast cancer.

### 2.2 FOXP3

**Figure 2:**
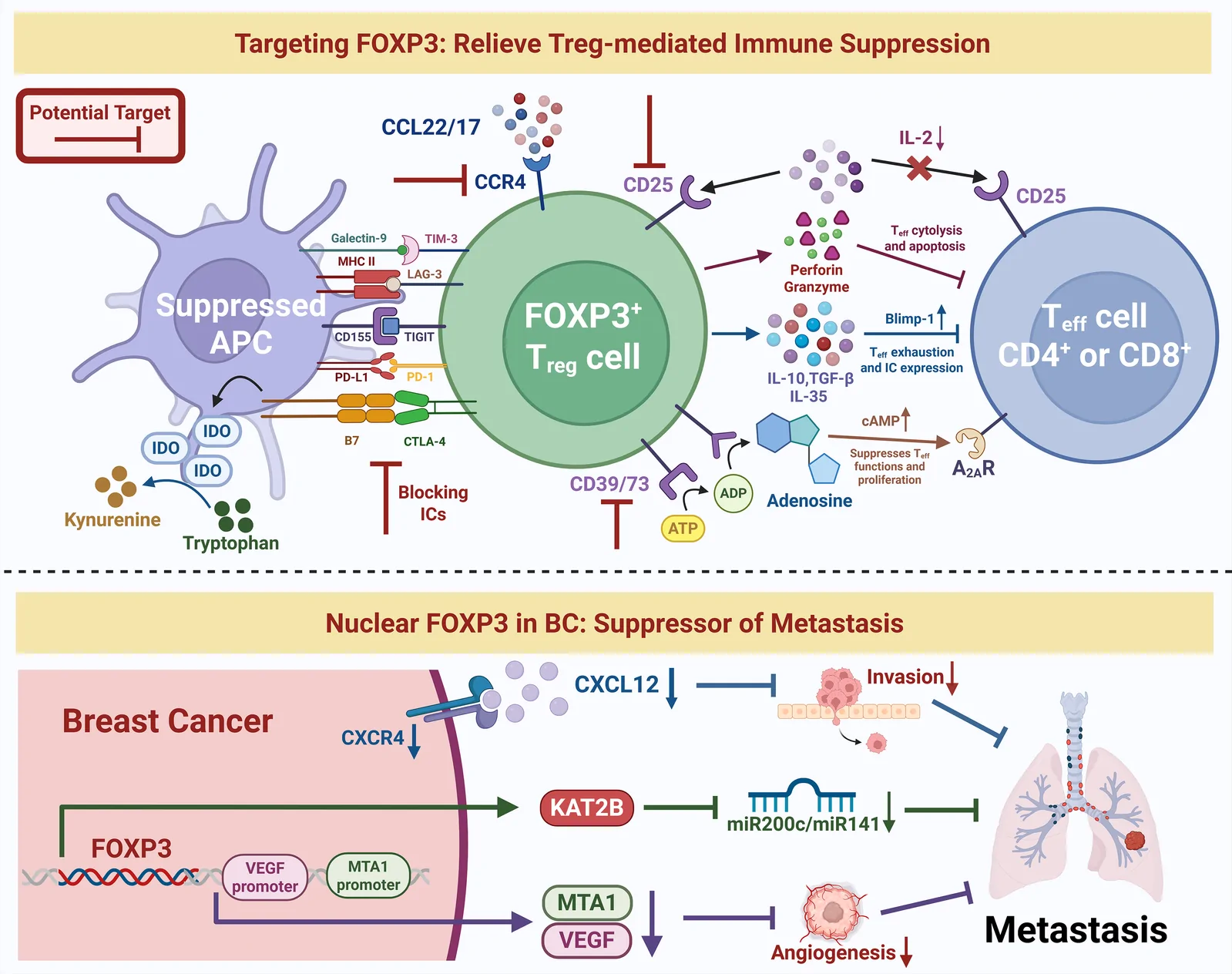
Dual roles of FOXP3 in breast cancer. FOXP3+ regulatory T cells establish an immunosuppressive tumor microenvironment through checkpoint-mediated inhibition, CD25-dependent IL-2 deprivation, secretion of IL-10, TGF-β, and IL-35, IDO-driven tryptophan catabolism, and CD39/CD73-mediated adenosine generation. By contrast, tumor-cell-intrinsic nuclear FOXP3 is associated with suppression of metastatic and angiogenic programs through regulation of MTA1, VEGF, CXCR4/CXCL12, and KAT2B–miR-200c/miR-141 signaling. These dual functions indicate that FOXP3-directed strategies should distinguish Treg-associated immune suppression from tumor-cell-intrinsic suppressive activity. Treg, regulatory T cell; Teff, effector T cell; IDO, indoleamine 2,3-dioxygenase.

FOXP3 is a member of the forkhead transcription factor family and is primarily expressed by a subset of CD4^+^ T cells commonly known as CD4^+^CD25^+^ regulatory T-cells (Tregs), which is important for Treg differentiation and function (85). FOXP3 has garnered significant attention recently due to its role in the generation of immune suppressor T cells, promoting the immune escape of tumor cells (86). The general mechanisms and agents targeting FOXP3 in breast cancer is shown in **Figure 2**. Aggressive breast cancer often correlates with abnormal expression of FOXP3, leading to dysregulation of oncogenes involved in metastasis. FOXP3 may also regulate chemokine receptor expression, which could explain the chemokine-driven and tissue-specific spread of many cancers (87). However, increasing evidence suggests that FOXP3 can also be expressed in tumor cells, including those in breast cancer (88–91). Notably, FOXP3 expressed in tumor cells functions differently or even oppositely in various tumors, indicating that the role of FOXP3 in the TME may be more complex than previously thought. The prognostic role of FOXP3 in breast cancer is controversial due to the influence of various factors such as molecular subtype and the location of Tregs (92). For example, studies indicate that FOXP3+ TILs are associated with improved overall survival (OS) in TNBC (93). Additionally, high FOXP3 mRNA levels contribute positively to OS and correlated with the efficacy of antitumor drugs (94). Moreover, a bioinformatic analysis suggests that FOXP3 inhibits breast cancer metastasis by downregulating the expression of metastasis-associated gene 1 (MTA1) (42). However, another meta-analysis suggests that a higher level of FOXP3+ TILs in patients with breast cancer contributes to poor OS (95). Furthermore, low levels of FOXP3+ TILs are correlated with a better prognosis for ER-positive breast cancer (96).

The expression of the FOXP3 gene is predominantly observed in Tregs and is subject to intricate regulatory mechanisms that govern its transcription and function. For instance, FOXP3 expression can be upregulated by pro-inflammatory cytokines, such as TNF-α and TGF-β, promoting the differentiation and activation of Tregs (97, 98). The induction and generation of FOXP3 are also governed by pathways such as Hedgehog, Notch1 (88). In the Hedgehog pathway, Hedgehog protein encourages naive CD4^+^ T cells to differentiate into Treg by upregulating FOXP3 expression (99). Furthermore, in the Yes associated protein (YAP)-dependent pathway, changes in FOXP3 expression in Tregs significantly facilitate tumor development and progression (100). Additionally, in cooperation with nuclear factor of activated T-cells (NF-AT), FOXP3 regulates the expression of lymphocyte-activation gene 3 (LAG-3), a CD4-related molecule and mediates tumor immunity (43). Another study shows that CD44 induces FOXP3 expression in Tregs and contributes to better prognosis in breast cancer (44).

Tregs expressing FOXP3 are instrumental in modulating the tumor microenvironment, facilitating immune evasion through the suppression of anti-neoplastic immune responses. Consequently, this immunosuppressive activity contributes to the promotion of tumor progression and the acquisition of metastatic potential (101–103). The suppressive function of FOXP3+ Tregs may be mediated through interactions with other immune cells. Binding of CTLA-4 on Tregs to CD80/CD86 on DCs delivers an inhibitory signal to effector T cells (104). Additionally, the interaction of lymphocyte activation gene on Tregs with MHC-II molecules on DCs lead to the same result. FOXP3+ Tregs can express immune inhibitory molecules on their surface (105). Among these, Tregs can express the IL-2 receptor subunit CD25(IL-2 receptor α-chain), which may lead to depletion and high consumption of the pro-inflammatory factor IL-2 in TME to suppress the growth of Teff cells (106). CD39 and CD73 can also be expressed on the cell surface, where they degrade extracellular ATP into adenosine, leading to the functional immunosuppression of target cells (107). FOXP3+ Tregs may also secrete immunosuppressive factors such as TGF-β and IL-10 to inhibit T cells, NK cells, and antigen-presenting cells (APCs) (108, 109).

The expression of the FOXP3 transcription factor in breast cancer cells is a subject of ongoing investigation, with its regulatory mechanisms being multifaceted and not yet fully elucidated (88). In Michigan Cancer Foundation–7 (MCF-7) breast cancer cells, the overexpression of FOXP3 increases the level of UBC9 mRNA, demonstrating FOXP3 as a novel transcriptional activator of the human UBC9 gene (110). Although FOXP3 has a dual role in breast cancer within TME, it mainly functions as a tumor suppressor gene in breast cancer cells and correlated studies are listed below. FOXP3 directly interacts with the VEGF promoter via specific forkhead-binding motifs to suppress its transcription. Notably, the inhibitory effects of FOXP3 in the supernatant on tube formation and sprouting in human umbilical vein endothelial cells (HUVECs) could be reversed by adding VEGF *in vitro (*45). Moreover, FOXP3 is identified as an X-linked breast cancer suppressor that represses the HER-2 oncogene, thereby inhibiting the proliferation of breast cancer cells (90). Present findings indicate that by activating the Wingless/Int-1 (Wnt)/beta−catenin signaling pathway, lysosomal protein transmembrane 5 (LAPTM5), which is negatively regulated by FOXP3, promotes the malignant phenotypes of breast cancer (46). Additionally, FOXP3 can directly interact with and repress the oncogene SKP-2 promoter. siRNA silencing of the FOXP3 gene in human mammary epithelial cells is proved to increase SKP2 expression (111). FOXP3 may help maintain normal breast epithelial characteristics by regulating the oncogene Zinc Finger E-Box Binding Homeobox 2 (ZEB2), and loss of FOXP3 in breast cancer cells results in deregulation of ZEB2, leading to progression the cancer (47). Furthermore, cytoskeleton-associated protein 2-like (CKAP2L), a mitotic spindle protein downregulated by FOXP3, can activate AKT/mTOR pathway and promote the progression of breast cancer, proving the protective value of FOXP3 in breast cancer (48). FOXP3 can also promote the apoptosis by upregulating the expression of programmed cell death 4 (PDCD4), functioning as a tumor suppressor gene (112).

In the context of metastasis, multiple studies conclude that the expression of FOXP3 serves as a suppressor of breast cancer metastasis. FOXP3 can directly bind to the promoter of CD44 and inhibit its protein expression, thereby suppressing the adhesion and invasion of human breast cancer cells (113). Additionally, FOXP3 downregulates the expression of MTA1 in breast cancer cells, affecting their ability of breast cancer cells to invade and metastasize *in vitro* and *in vivo*, thereby inhibiting breast cancer metastasis (113). Furthermore, the levels of miR-200c and 141 were lower in low-FOXP3 breast cancer cells compared to those high-FOXP3 breast cancer cells, indicating that the FOXP3-Lysine Acetyltransferase 2B (KAT2B) axis regulates microRNA-200c and 141, which are associated with tumor metastasis in breast cancer (114). Moreover, FOXP3 may also regulate the expression of chemokine receptor CXCR4, which regulates the development of breast cancer by stimulating cell migration toward CXCL12-expressing sites of metastatic spread (49, 115). Another study shows that endogenous Runt-Related Transcription Factor 1 (Runx1) and FOXP3 physically interact in normal mammary cells, blocking Runx1 transcriptional activity. Reduced Runx1 transcriptional activity decreases tumor cell migration properties, indicating that FOXP3 acts as an inhibitor for tumor cell migration (50).

FOXP3 may serve as a potential immune checkpoint biomarker in breast cancer (116, 117). Tumor cells can actively recruit and induce Tregs to block innate and adaptive immune response, effector function and memory response, which can inhibit the efficacy of therapeutic cancer vaccines. Depletion of Tregs before or early in tumor development contributes to complete tumor eradication (118–120). Additionally, Treg reactivation induced by immune checkpoint inhibitors may contribute to acquired resistance to treatment (121). However, the role of FOXP3 in cancer depends on the research methods, the type of cells expressing FOXP3, and the stages and molecular subtypes of breast cancer patients. Thus, a larger number of studies is warranted to elucidate the mechanisms of FOXP3 functions.

### 2.3 WT1

**Figure 3:**
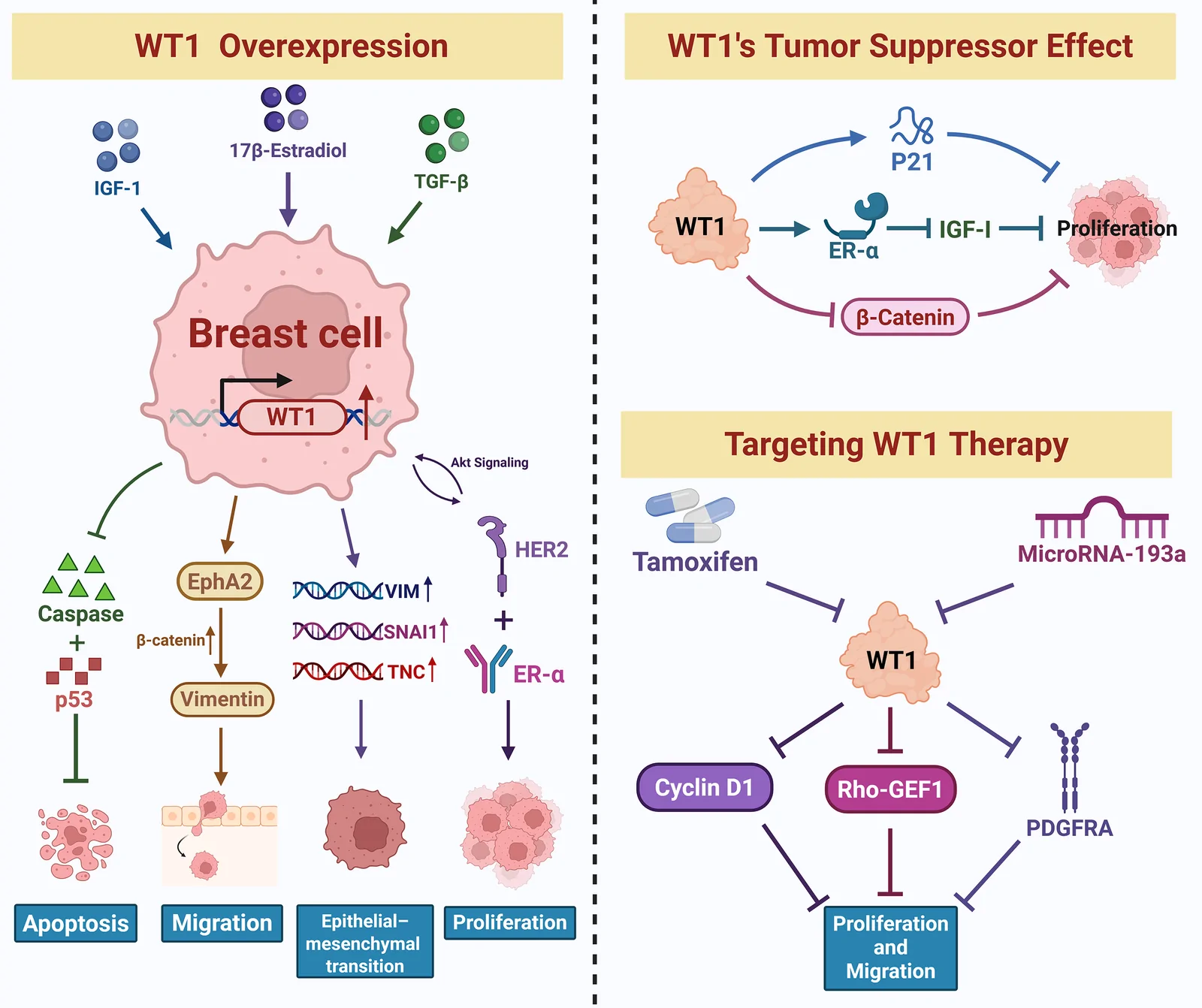
WT1-associated signaling networks and therapeutic implications in breast cancer. WT1 expression in breast cancer is shaped by growth factor, hormonal, and oncogenic signaling, including IGF-1, 17β-estradiol, TGF-β, HER2, ER-α, and Akt-related pathways. WT1 overexpression can promote malignant progression by regulating apoptosis resistance, migration, epithelial–mesenchymal transition, and proliferation through p53/caspase, EphA2–vimentin, EMT-related genes, and HER2/ER-α-linked signaling. Conversely, WT1 may exert tumor-suppressive effects through p21, ER-α/IGF-I, and β-catenin pathways. These context-dependent activities support WT1 as both a functional biomarker and a candidate therapeutic target in breast cancer. EMT, epithelial–mesenchymal transition; ER-α, estrogen receptor α; HER2, human epidermal growth factor receptor 2; IGF-1, insulin-like growth factor 1.

WT1 is a transcription factor that regulates the epithelial-mesenchymal balance during embryonic development. In normal tissues, WT1 is expressed in the gonads, uterus, kidneys, mesothelium, and hematopoietic progenitor cells. Through DNA binding, WT1 can regulate transcription according to the context of the DNA-binding sites or the cell type in which it is expressed (122). Recent studies indicate that WT1 can also be expressed in several tumor types and is highly expressed and hypermethylated in breast cancer (123–126). WT1 was originally defined as a tumor suppressor (127). For example, a study shows that different tumors may share a common WT1-related defect resulting in altered regulation of target genes, indicating that WT1 as a tumor suppressor (128). Furthermore, WT1 plays an important role in maintaining the normal growth of mammary epithelial cells and dysregulated WT1 expression may contribute to breast cancer development (129). However, multiple studies later indicate that WT1 is indeed an oncogene rather than a tumor suppressor gene (130, 131). For instance, WT1 has been shown to promote non-small cell lung cancer (NSCLC) cell proliferation by upregulating cyclin D1 and phosphorylated retinoblastoma protein (p-pRb) expression (132). Additionally, high WT1 mRNA expression correlates with basal-like and HER2 molecular subtypes and poor prognosis in breast cancer (133, 134). Thus, WT1, which influences tumor development and angiogenesis, has become a promising therapeutic target in breast cancer (135). **Figure 3** delineates the dual roles of WT1 in breast cancer, acting as either an oncogene or a tumor suppressor, and also highlights potential WT1-targeted therapeutic strategies.

WT1 expression is subject to a variety of regulatory mechanisms, with its levels being intricately linked to those of HER2 and the epidermal growth factor receptor (EGFR). Notably, the presence of WT1 is often observed in conjunction with elevated expression of ER-α and HER2, which can drive cellular proliferation and may play a role in the evolution and advancement of cancer (136). Moreover, HER2 can engage Akt to increase WT1 expression, with WT1 protein playing a vital role in regulating cell cycle progression and apoptosis in HER2-overexpressing breast cancer cells (137). Additionally, WT1 functions through the activation of the signaling pathways mediated by the members of the EGFR family, as demonstrated by the upregulation of EGFR and HER2, increased estrogen-independent growth and anti-estrogen insensitivity of WT1-transfected MCF-7 cells (138, 139). Other mechanisms have also been revealed that could be possible therapeutic methods in the future. For example, WT1 has been shown to be an important early target of progestins, regulating both proliferation and differentiation in breast cancer cells (140). Moreover, microRNA-193a can inhibit the proliferation and metastasis of breast cancer by downregulating WT1, indicating that restoring microRNA may be a possible therapy for breast cancer (141). Furthermore, WT1 can be employed in keratinocyte growth factor (KGF)-mediated signaling, regulating breast cancer cell proliferation and motility, which may represent therapeutic targets for the prevention of breast cancer progression (51). Additionally, the WT1-zinc finger domain (ZF) plasmid negatively regulates endogenous WT1 in breast cancer cells, providing a potential candidate for gene therapy in breast cancer (142). Another study indicates that in various types of solid cancer, downregulated tumor suppressor microRNA-125a can target zinc finger and BTB domain containing 7A gene (Zbtb7a), (143). Scientists also found that New York esophageal squamous cell carcinoma 1 (NY-ESO-1) and WT1 genes were overexpressed in TNBC, suggesting a possible relation between these two genes (125).

WT1 orchestrates cellular proliferation and the metastatic spread of breast cancer by modulating the activity of downstream molecular effectors. This intricate regulatory network involves the activation or fine-tuning of various signaling pathways, thereby enhancing the malignant potential of breast cancer cells (144). For breast cancer cell development, WT1 can regulate the expression of stromal interaction molecule 1 (STIM1), an essential regulator of Ca^2+^ entry in non-excitable cells, which is linked to cancer development (52). Additionally, miR-361-5p expression is inversely correlated with WT1 and may be associated with poor prognosis while serving as a potential non-invasive biomarker for early breast cancer detection (145). Moreover, WT1 inactivation in tumor cell may result in deregulated insulin-like growth factor I receptor (IGF-IR) gene expression, with IGF-IR playing an important role in breast cancer development and progression (146–148). Others have revealed that when down-regulation of WT1 is impaired by undetermined causes, WT1-expressing progenitor cells continue to proliferate and eventually transform into tumor cells as a result of the accumulation of unknown genetic events (123). Regarding drug resistance, WT1 gene expression correlates with CYP3A4 levels and is associated with poor chemotherapy response, leading to poor prognosis potentially due to cancer-related epithelial-to-mesenchymal transition (EMT) (149, 150). Moreover, a genome-wide association study identified a WT1 variant with better response to 5-fluorouracil, pirarubicin and cyclophosphamide NACT in breast cancer patients (151, 152). Furthermore, scientists found the relationship between the 36-38 kDa isoform of WT1 and the absence of ER function in advanced breast cancer, explaining the resistance of drugs targeting ER in the late stage of breast cancer (153). WT1 is also defined as a mediator of antiestrogen resistance in breast cancer through the down-regulation of ERα expression. WT1 protein can bind to endogenous WT1 consensus sites in the proximal promoter of Erα, thus inhibiting the transcriptional activity of the ERα promoter in a WT1 site sequence-specific manner, indicating WT1 inhibitors as a potential means of restoring antiestrogen responsiveness in breast cancer therapy (154). Research has consistently pointed to the multifaceted regulatory network of WT1 in the context of breast cancer. Specifically, WT1 downregulates the β-catenin/TCF signaling pathway by destabilizing beta-catenin, a critical mediator of cell proliferation and oncogenic processes (155). All isoforms of WT1 directly activate the promoter activity of the ER-α66 gene while suppressing ER-α36 promoter activity, implicating WT1 as one of the coordinators that orchestrate genomic and non-genomic estrogen signaling (156).

Based on the discovery of WT1 overexpression in breast cancer cells, WT1-specific cytotoxic T lymphocytes (CTLs) have become a promising target against breast cancer (157). However, the induction of autologous WT1-specific CTLs may confer only modest protection against tumor growth. Strategies that allow a high level of peptide/MHC complex presentation are thus required (158, 159). In this regard, scientists have developed a range of therapeutic vaccines specifically targeting the WT1 antigen. There are mainly two categories of vaccines: WT1-peptide vaccine and WT1-targeted DC vaccine (160). The WT1-peptide vaccine typically consists of specific peptides derived from the WT1 protein. Once injected, the WT1 peptides are taken up by APCs, including DC. These cells process the peptides and present them on their surface using MHC molecules, which can be recognized by CD8+ CTLs and CD4+ helper T cells, leading to the activation and expansion of these T cell populations, which specifically target breast cancer cells expressing the WT1 antigen (161). Activated CD8+ T cells can recognize and kill breast cancer cells expressing the WT1 antigen (162). Helper T cells enhance the immune response through cytokine release, promoting the activation of other immune cells. Due to its overexpression in breast cancer cells and the limited expression in normal tissues, the likelihood of AEs resulting from immunotherapies targeting WT1 is reduced (163). The WT1-targeted DC vaccine, however, is a more complex immunotherapy (164). As previously discovered by scientists, DCs are professional APCs that have been deployed as cellular vaccines due to their ability to induce anti-cancer immunity and bridge adaptive and innate immunity (165). After being harvested from the patient, DCs are then cultured *in vitro* and loaded with WT1 peptides or whole WT1 protein (166, 167). The loading process typically includes the use of cytokines or other factors to mature the DCs, enhancing their ability to present antigens and stimulate T cells. The activated, antigen-loaded DCs are then re-infused back into the patient. Once in the body, these DCs migrate to lymph nodes, where they present the WT1 antigen to naive T cells, leading to the robust activation of both CD4+ and CD8+ T cells (168). This results in a more extensive and sustained immune response against WT1-expressing cancer cells (169).

Therefore, WT1 represents a promising target to inhibit the proliferation and metastasis of breast cancer cells and to decrease drug resistance. DC-based vaccines, leveraging host immune system, may elicit a more robust and enduring immune response. In contrast, peptide vaccines offer a more simplified and manufacturable approach. Although clinical trials for WT1-targeting therapeutic vaccines have demonstrated promising results in solid tumor contexts, further studies are warranted to establish their efficacy and safety profiles (170, 171).

## 3 Neoadjuvant immunotherapies in breast cancer

Ongoing research aims to address these issues and improve patient selection for neoadjuvant immunotherapy. The success of clinical trials, such as KEYNOTE-522, which led to the FDA approval of pembrolizumab in combination with chemotherapy for early-stage TNBC, underscores the potential of this approach in breast cancer. **Supplemetary Tables 2**, **3** list the ongoing and reported clinical studies assessing the efficacy of neoadjuvant immunotherapy in TNBC.

### 3.1 Drugs targeting PD-1/PD-L1

Immunotherapies that target PD-1/PD-L1 axis have achieved unprecedented success in a wide variety of cancers by enhancing cancer immunotherapy, including lung cancer, renal cancer and breast cancer (182–186). Immunotherapeutic strategies encompass agents such as avelumab, durvalumab, and atezolizumab, which target the PD-L1 pathway, as well as pembrolizumab, which directly targets PD-1 (187). Studies on PD-1 inhibitors, including camrelizumab, nivolumab, and spartalizumab, along with the PD-L1 inhibitor FAZ053, have been well documented, albeit with less frequency compared to the aforementioned agents (188, 189). These drugs are effective and durable for some patients diagnosed with breast cancer. However, side effects and toxicity exist and the prognosis of patients is related to factors such as the type of immunosuppressants, tumor stage, tumor molecular type, age, and gender (190, 191). We herein summarize ongoing or completed clinical studies on major neoadjuvant PD-1/PD-L1 immunotherapies in breast cancer.

#### 3.1.1 Atezolizumab

Atezolizumab is the first immune checkpoint inhibitor which receives a breast cancer-specific approval according to the results based on IMpassion130 (192–194). It was also widely evaluated in NSCLC (195, 196). However, controversy remains on the effect of atezolizumab as neoadjuvant immunotherapy in breast cancer patients, with some research reporting positive results while some drew negative conclusions.

In IMpassion130 (NCT02425891), a phase 3 trial including 902 patients over 18 years old with TNBC, showed that atezolizumab plus nab-paclitaxel benefited both the intention-to-treat (ITT) population and the PD-L1–positive subgroup. Scientists randomly assigned patients in a 1:1 ratio to receive atezolizumab plus nab-paclitaxel or placebo plus nab-paclitaxel. All AEs were consistent with those previously observed with the medication. Using progression-free survival (PFS) and OS as primary endpoints and comparing trial groups through common methods such as Cox proportional-hazards model or Kaplan–Meier analysis, they found that PFS was significantly longer in the atezolizumab/nab-paclitaxel group than in the placebo/nab-paclitaxel group (median, 7.2 months vs. 5.5 months; P = 0.002). Meanwhile, OS was also longer in the atezolizumab/nab-paclitaxel group than in the placebo/nab–paclitaxel group (median, 21.3 months vs. 17.6 months). Notably, the OS benefit in the ITT population was not statistically significant while OS benefit was clinically meaningful in patients with PD-L1 immune cell-positive disease. Although IMpassion130 evaluated unresectable locally advanced or metastatic TNBC rather than neoadjuvant treatment, it provided important background evidence for atezolizumab in breast cancer (197–199). In IMpassion031(NCT03197935), a phase 3 trial with pCR as the primary endpoint, neoadjuvant atezolizumab plus nab-paclitaxel and anthracycline-based chemotherapy significantly improved pCR (173, 200). In another study NCI10013 (NCT02883062), scientists found that atezolizumab combined with neoadjuvant carboplatin and paclitaxel appeared to have had a statistically significant and clinically relevant increased pCR rate in patients with stages II and III TNBC (175, 201). Additionally, in patients with anthracycline-resistant early-stage TNBC, the efficacy of neoadjuvant atezolizumab and nab-paclitaxel was evaluated in a 5-year, phase II study (NCT02530489). The research met its primary endpoint pCR/residual cancer burden class I (RCB-I) (46% vs 5%), demonstrating that the combination of therapy has promising effect in this high-risk population (180).

In contrast, clinical trials like IMpassion131 and IMpassion050 have reported varying outcomes (202, 203). In the NeoTRIP Michelangelo randomized study (NCT02620280), 280 TNBC patients were randomly assigned to receive nab-paclitaxel 125 mg/m2 IV on days 1 and 8, either with atezolizumab 1200 mg on day 1 (n=138) or without (n=142). By ITT analysis, pCR rate after treatment with atezolizumab did not reach statistical significance compared to placebo (48.6% vs.44.4%; P = 0.48), indicating that atezolizumab may not be an option for neoadjuvant therapy (172). Additionally, in phase III IMpassion131 trial (NCT03125902), scientists took investigator-assessed PFS as primary endpoint and evaluated atezolizumab plus paclitaxel as a first-line therapy for TNBC. The trial ended up with the conclusion that neither PFS or OS was improved by atezolizumab combined with paclitaxel (202).

As previously discussed, clinical studies have demonstrated that the combination of atezolizumab with chemotherapy is associated with benefits and an acceptable safety profile, suggesting that the concomitant use of atezolizumab and chemotherapy may be a viable treatment approach (204). In addition to atezolizumab combined with nab-paclitaxel, the effect of atezolizumab plus anthracycline-based chemotherapy in patients was also tested. In a randomized, double-blind phase 2b ALICE trial (NCT03164993), patient with metastatic TNBC received pegylated liposomal doxorubicin (PLD) and low-dose cyclophosphamide plus atezolizumab or placebo. The addition of atezolizumab was well tolerated and significantly improved the PFS and progression-free proportion in breast cancer patients (205). Another open-label, phase I study (NCT02605915) enrolled 73 patients to evaluate the efficacy of atezolizumab with trastuzumab/pertuzumab, atezolizumab with the antibody-drug conjugate T-DM1, or atezolizumab with trastuzumab/pertuzumab and docetaxel, indicating that atezolizumab with antibody-dependent cellular cytotoxicity or antibody-drug conjugate agents appears safe (206).

Why do atezolizumab trials produce such different results? First, nab-paclitaxel (IMpassion130 and IMpassion031) may enhance immune priming through albumin-mediated transcytosis, whereas solvent-based paclitaxel (IMpassion131) requires corticosteroid premedication and lacks this property (207–209). Second, the SP142 assay used in IMpassion130/131 and NeoTRIP has lower sensitivity than the 22C3 assay used in KEYNOTE-522, and tissue handling differences between core biopsy and surgical specimen may alter PD-L1 dynamics (210). Third, chemotherapy backbones and treatment schedules differed substantially. NeoTRIP used neoadjuvant carboplatin plus nab-paclitaxel, with anthracycline-based chemotherapy administered after surgery, whereas IMpassion031 incorporated sequential nab-paclitaxel and anthracycline/cyclophosphamide entirely within the neoadjuvant regimen. These differences may have contributed to the divergent outcomes. Fourth, endpoints capture different biological dimensions. pCR reflects early immune engagement within the tumor, while PFS/EFS reflect sustained systemic immune memory (211, 212). Finally, timing and duration of immunotherapy exposure vary. Concurrent administration with chemotherapy may maximize immunogenic cell death, but the optimal duration in the neoadjuvant setting remains unclear (213). These factors highlight why cross-trial comparisons should be made cautiously, and why standardized biomarker assays and harmonized trial designs are needed.

In summary, while numerous trials have been conducted to assess the efficacy of atezolizumab in combination with chemotherapy or immunotherapy, only select regimens, such as atezolizumab in conjunction with nab-paclitaxel, are utilized in clinical practice for patients with unresectable locally advanced or PD-L1-positive metastatic TNBC (214, 215). The evaluation of atezolizumab combined with nab-paclitaxel in certain chemotherapy-resistant patients has yielded promising results. However, results vary across trials due to differences in chemotherapy backbone, PD-L1 assay methodology, patient populations, and endpoints. Further work is needed to identify optimal drug combinations and biomarker-driven patient selection strategies in this setting.

#### 3.1.2 Pembrolizumab

Pembrolizumab has changed the neoadjuvant treatment landscape for TNBC (216–219), particularly since the FDA approval of the KEYNOTE-522 regimen (220–223). Clinical studies with the same proved conclusions and new findings are introduced as below.

In KEYNOTE-522 trial (NCT03036488), 1174 patients with previously untreated stage II or III TNBC were assigned in a 2:1 ratio to receive four cycles of pembrolizumab (200mg/dose) every 3 weeks in addition of paclitaxel and carboplatin or placebo every 3 weeks plus paclitaxel and carboplatin as neoadjuvant therapy. Two groups of patients above then received an additional four cycles of pembrolizumab or placebo (224). Patients of both groups were treated the same after definitive surgery. pCR at the time of definitive surgery and event-free survival (EFS) in ITT population were taken as primary end points. Through primary analysis of pCR, scientists found that pCR rate in pembrolizumab–chemotherapy group was higher than placebo–chemotherapy group (64.8% vs. 51.2%, P<0.001) (224). Additionally, estimated EFS at 36 months was significantly higher in the pembrolizumab–chemotherapy group compared with the placebo–chemotherapy group (84.5% vs. 76.8%) (225). These findings also demonstrated that pembrolizumab improved EFS among most patients absent of pCR (211). In phase 2 I-SPY2 trial (NCT01042379) including 250 women, Participants in the control arm received standard NACT while participants in the pembrolizumab arm received standard NACT in addition of 200 mg pembrolizumab every 3 weeks for 4 cycles together with paclitaxel. Taking pCR rate as endpoint, they found that estimated pCR rates for HER2-negative, HR-positive/HER2-negative, and TNBC signatures in the pembrolizumab arm were higher than control arm (44% vs. 17%, 30% vs. 13%, 60% vs. 22%, respectively), which indicated the same as the study above that the addition of pembrolizumab to standard NACT was effective (212, 226, 227). Three-year follow-up analysis, including participants with follow-up data available as of February 26, 2019, showed that achieving pCR after neoadjuvant therapy improved long-term outcome, making the therapy a “punch combinations” for breast cancer patients (228).

In other trials with fewer participants, opinions on the benefit of pembrolizumab plus standard NACT stay positive and some new findings have also been reported (176, 177, 229). In KEYNOTE-173 study (NCT02622074), 60 patients were enrolled and recommended phase II dose was one of the primary end points. Through the similar controlled process, scientists came with the same conclusion that NACT and pembrolizumab for high-risk, early-stage TNBC showed promising antitumor activity. Additionally, by digging into exploratory end points, positive correlation between pCR rate and tumor PD-L1 expression was found, which may guide experiment and treatment in the future (177). In a single-arm phase II trial (AGO-B-041/NCT03289819), similar results were reported that neoadjuvant nab-paclitaxel or anthracycline in addition of pembrolizumab showed an encouraging pCR rate (66%). However, during the process, scientists found that a pre-chemotherapy pembrolizumab dose did not indicate different pCR rates, which suggested that different orders of treatment might lead to different outcomes (176). The ongoing PELICAN-IPC 2015-016/Oncodistinct-003 (NCT03515798) took pCR rate in overall population as the primary endpoint, whose results have not been reported yet (230).

In conclusion, clinical trials have consistently shown that the combination of pembrolizumab with NACT is both effective and safe for treating TNBC. Notably, the KEYNOTE-522 trial has established a new standard of care, demonstrating significantly improved pCR rates and EFS. Moreover, emerging data from smaller studies and alternative treatment combinations, including HER2 inhibitors and radiotherapy, suggest promising advancements in targeted cancer therapies. This progress underscores the potential of immunotherapy to transform the landscape of breast cancer treatment.

#### 3.1.3 Durvalumab

Durvalumab, a PD-L1 inhibitor, has been extensively studied in neoadjuvant therapy across a spectrum of cancers, encompassing urothelial carcinoma, non-small-cell lung cancer, and breast cancer (231). In 2022, GeparNuevo trial (NCT02685059), which suggested that the addition of durvalumab to NACT increased the pCR rate for patients with TNBC, has set the foundation for studies in this area and is as foundational as the KEYNOTE-522 trial and IMpassion130 trial in their own area (178, 200). The details of the GeparNuevo trial and other related trials are introduced below.

As a randomized phase II double-blind placebo-controlled study, a total of 174 patients with TNBC were enrolled in GeparNuevo. Patients were administered durvalumab or placebo every four weeks combined with nab-paclitaxel followed by standard epirubicin and cyclophosphamide, and durvalumab/placebo alone was given 2 weeks before nab-paclitaxel in the window-phase. With pCR as primary objective, researchers found that pCR rate with durvalumab was 53.4% (95% CI 42.5% to 61.4%) versus placebo 44.2% (95% CI 33.5% to 55.3%) and the effect of durvalumab was seen only in the window arm (pCR 61.0% versus 41.4%, P = 0.035), which led to the conclusion that durvalumab together with anthracycline-/taxane-based NACT increased pCR rate particularly in patients given durvalumab alone in the window-phase. Thyroid dysfunction of any grade was the most common immune-related AE accounting for 47% (178, 232). According to further studies, significant improvement of secondary endpoints invasive disease-free survival (iDFS), distant disease-free survival (DDFS) and OS was observed. Despite a moderate pCR increase and absence of adjuvant therapy, Durvalumab added to NACT in TNBC significantly improved survival (233).

Other clinical trials yielded the similar results that the addition of durvalumab benefited patients with breast cancer. In the phase II I-SPY2 trial of stage II/III HER2-negative breast cancer (NCT01042379), 73 patients were given durvalumab and poly-ADP-ribose polymerase (PARP) inhibitor olaparib combined with standard paclitaxel NACT (DOP) while other 299 patients were given standard of care (paclitaxel) control. Although more patients in the DOP group underwent immune-related grade 3 AEs (12.3% versus 1.3%), DOP had superior advantages over standard NACT in HER2-negative breast cancer, particularly in a highly sensitive subset of high-risk HR-positive patients (234, 235). In a trial (NCT02489448) evaluating the effect of durvalumab concurrent with neoadjuvant nab-paclitaxel and dose-dense doxorubicin/cyclophosphamide chemotherapy, researchers found the result of 44% pCR rate, indicating the efficacy of neoadjuvant durvalumab in breast cancer (236, 237).

Several theories have been reported behind the effect of neoadjuvant durvalumab. By examining predictive markers of response, scientists found that the TGF-β pathway was associated with immune-poor phenotype and residual disease in basal-like TNBC under the treatment of neoadjuvant durvalumab combined with chemotherapy (238). An analysis from the same phase I/II clinical trial (NCT02489448) indicates that expression of PD-L1 in neoplasm cells and immune cells in stroma contributes to higher rates of pCR to durvalumab together with chemotherapy in patients with TNBC (239). Although the incidence of TNBC is higher in African American (AA) women, pCR rates, 3-year OS and EFS under neoadjuvant durvalumab combined with chemotherapy are comparable to those in non-AA women (240).

In summary, durvalumab, a PD-L1 inhibitor, has shown promise in neoadjuvant therapy for breast cancer, notably TNBC, as demonstrated by the GeparNuevo trial, which reported increased pCR rates and improved survival outcomes. While trials like I-SPY2 confirmed these benefits, they also noted a higher incidence of immune-related AEs. The efficacy of durvalumab is linked to TGF-β pathway involvement and PD-L1 expression, showing consistent benefits across different demographics. These findings highlight durvalumab’s potential in enhancing NACT for breast cancer.

#### 3.1.4 Avelumab

Avelumab is a human anti-PD-L1 monoclonal antibody that inhibits the interaction between PD-1 and PD-L1, similar to the mechanism of atezolizumab (241). Studies investigating the efficacy of avelumab as neoadjuvant therapy in breast cancer are limited. However, there are still several studies demonstrating its efficacy in breast cancer treatment (242). The process and mechanisms of ongoing trials and discussion of why studies in this area are limited are listed below.

Several mechanisms have been identified as pivotal in the treatment of breast cancer. Avelumab significantly enhanced NK-cell mediated cytotoxicity against TNBC cells and tumor cells expressing higher levels of PD-L1 were more sensitive to Avelumab-mediated antibody-dependent cell-mediated cytotoxicity (ADCC) (243). In an *in vitro* study, IL-2 and IL-15 from NK cells can increase Avelumab-triggered cytokine production and degranulation together with increased lytic activity against tumor cells, suggesting that avelumab plus immunomodulators might be a solution to increase the efficacy of avelumab in breast cancer (244). Moreover, cyclin-dependent kinase (CDK) inhibition at suboptimal doses promotes immune cell recruitment to tumors and increases PD-L1 expression by surviving TNBC cells, which has been shown to reduce tumor growth in mice together with avelumab (245).

The limitations of avelumab in treatment can be attributed to the following factors: Firstly, pembrolizumab and atezolizumab were both discovered earlier and were approved by FDA for use in a wide range of indications, while the effectiveness of avelumab against cancer was first proved by JAVELIN 100 trial (NCT02603432) in 2020 (246, 247). Secondly, the effect of avelumab to induce ADCC of tumor cells and enhance anti-tumor efficacy by modulators has been emphasized *in vitro* studies, suggesting it may have an enhanced mechanism of anti-tumor activity (243, 248, 249). However, clinical data have not yet established whether these mechanisms contribute meaningfully to avelumab activity in patients with breast cancer. Consequently, a distinct clinical advantage over other checkpoint inhibitors has not been demonstrated (250). Additionally, unlike other anti-PD-1 antibodies, which target T-cells, avelumab targets tumor cells, and is supposed to have fewer side effects, including a lower risk of immune-related AEs. However, a case of radiotherapy with avelumab lead to acute toxicities in TNBC has been reported, arousing the problem of unknown side effects (251). Additionally, in the area of breast cancer, pembrolizumab and atezolizumab are approved for neoadjuvant treatment in breast cancer based on the results from KEYNOTE-522 trial and IMpassion130 trial, while the effectiveness of avelumab has not been proved yet.

As a newer entrant in the PD-1/PD-L1 inhibitor class, avelumab faces challenges due to the scarcity of foundational trials and a lack of clear superiority over existing agents, which makes it less favored in both preclinical and clinical research. Although preclinical studies suggest potential benefits of avelumab in enhancing NK-cell-mediated cytotoxicity and working with immunomodulators, clinical evidence is limited. Early clinical trials have shown modest efficacy and some acceptable safety profiles, but avelumab faces significant competition from better-established inhibitors like pembrolizumab and atezolizumab, which have broader FDA approvals and extensive clinical validation. This competitive landscape and limited distinct advantages hinder avelumab’s adoption in breast cancer treatment.

#### 3.1.5 Nivolumab

Nivolumab, a fully human monoclonal antibody that targets PD-1 receptor, has gained attention in the treatment of various cancers, including breast cancer (252, 253). In breast cancer, particularly TNBC, nivolumab has shown promising efficacy (252). However, severe side effects have also been reported (254).

A neoadjuvant phase II GIADA trial (NCT04659551) enrolled stage II-IIIA premenopausal patients with Luminal B-like breast cancer. Patients received three 21-day cycles of epirubicin/cyclophosphamide followed by eight 14-day cycles of nivolumab, with triptorelin starting concomitantly with chemotherapy and exemestane starting concomitantly with nivolumab. Taking pCR as the primary endpoint, patients with PAM50 basal breast cancer (50%) have a significantly higher pCR compared to other subtypes, which suggests that Luminal B-like breast cancers with a basal molecular subtype may respond well to sequential anthracyclines and nivolumab (188). In the open-label, randomized phase II OXEL study (NCT03487666), the immunologic effects of nivolumab, capecitabine and their combination were assessed based on changes in the primary immunologic score (PIS), which served as the primary endpoint. Involving 45 women with TNBC who had residual invasive disease after standard NACT, results demonstrated that the combination of nivolumab and capecitabine led to a significantly greater increase in PIS from baseline to week 6 (91%) compared to nivolumab alone (47%) or capecitabine alone (53%), with a log-rank p-value of 0.08, thus meeting the pre-specified primary endpoint (255). Apart from clinical trials, a preclinical study has also demonstrated the therapeutic efficacy neoadjuvant combination therapy (nivolumab plus bevacizumab). By engrafting the human immune system into severely immunodeficient mice and subsequently implanting TNBC cells into them, humanized mouse models were created and were treated with neoadjuvant combination therapy (bevacizumab combined with nivolumab). Through analysis of TME, the combination therapy significantly inhibited tumor growth and prevented recurrence and metastasis by normalizing tumor vessels and inducing CD8+ T cell infiltration and activation (256).

However, while the aforementioned studies have established the efficacy of Nivolumab in breast cancer, treatment-related AEs have also been documented. In the noncomparative phase 1b/2 CheckMate 7A8 trial (NCT04075604), neoadjuvant therapy with nivolumab, palbociclib, and anastrozole was assessed in ER+/HER2- breast cancer patients. Among the 21 participants, treatment was discontinued for 9 due to toxicity, which included grade 3/4 hepatic AEs (n = 6), grade 3 febrile neutropenia (n = 1), grade 1 pneumonitis (n = 1), and grade 3 rash with grade 2 immune-mediated pneumonitis (n = 1). Consequently, the study was closed early. The high incidence of grade 3/4 hepatotoxicity and treatment discontinuation indicates that this combination should not be further pursued for the treatment of primary ER+/HER2- breast cancer (257). Furthermore, a pilot phase I trial (NCT04185311) evaluated the efficacy of systemic nivolumab and ipilimumab and intratumor talimogene laherparepvec (T-VEC). With 6 patients involved, this immunotherapy regimen provided responses in patients with advanced or relapsed HER2-negative breast cancer, at the expense of long-term toxicities (258). Moreover, a case of radiation recall dermatitis with nivolumab in a woman with breast cancer was reported, suggesting unknown AEs may occur in the process of nivolumab treatment (254).

Nivolumab holds promise for breast cancer treatment, notably TNBC, yet it is associated with significant side effects. Clinical trials have demonstrated its potential efficacy, particularly in combination therapies, yet high toxicity levels have led to early termination in some studies. A thorough assessment of its AEs is warranted.

### 3.2 Drugs targeting FOXP3

FOXP3, a transcription factor that is mainly expressed by CD4+CD25+ regulatory T-cells, is essential in the function of Treg to inhibit the immune response of a host, allowing tumor cell to escape from immune system and leading to poor prognosis (259–262). However, recent findings indicate that elevated FOXP3 expression correlates with improved OS, contradicting previous conclusion (263). Thus, as previously mentioned, the role of FOXP3 as either a tumor suppressor or inducer remains to be elucidated (264). Beyond serving as a direct target for immune checkpoint inhibitors such as PD-1/PD-L1, FOXP3 may serve as a prognostic or predictive biomarker and as an investigational therapeutic target. Thus, FOXP3 is considered to be directly and indirectly targeted in neoadjuvant therapies in breast cancer. Herein we summarize the role of FOXP3 in breast cancer, possible drugs targeting FOXP3 and ongoing/completed clinical or preclinical trials.

The expression of FOXP3 is widely recognized as a prognostic marker in tumor remission, forming the foundational rationale for drugs targeting FOXP3 in various neoadjuvant treatment strategies (265, 266). Multivariate analysis identified FOXP3 intra-tumoral TILs (iTILs) expression status as an important independent factor contributing to poor prognosis (267). Moreover, patients of TNBC with a high CD8+/FOXP3+ ratio are more sensitive to anthracycline and taxane-based chemotherapeutic regimens, and the CD8+/FOXP3+ ratio in conjunction with Ki-67 could predict pCR following NACT in TNBC (268). In another study which evaluated 183 patients who underwent curative surgery for breast cancer, focusing on patient survival, it came out that the 10-year disease-specific survival (DSS) rate was 62.5% in FOXP3-strong-positive compared to 81.3% in FOXP3-negative or weak-positive patients, indicating that strong FOXP3 expression in breast cancer cells was associated with poor prognosis (93, 269, 270). By conducting expression studies at the mRNA level and protein level, researchers came up with the conclusion that the methylated FOXP3 cases that did not express protein were significantly associated with positive lymph node metastasis and HER-2 status, leading to poor prognosis (271).

However, in a study focusing on FOXP3 expression within cancer cells, patients with HER2-overexpressing breast cancer underwent NACT treatment. Using Kaplan-Meier analysis and Cox regression model to assess relapse-free and OS, scientists found that FOXP3 expression in breast cancer cells was associated with better relapse-free (P = 0.005) and OS (P = 0.03), indicating that the existence of FOXP3+ tumor cells might be an independent prognostic factor of improved outcome in HER2-overexpressing breast cancer (263). Another study indicated that FOXP3 on regulatory T cells was a significantly favorable marker in the IL-33 high group, which showed better prognosis (272). There were also studies which discovered no relationship between FOXP3+ TILs numbers and cancer progression in patients with synchronous bilateral breast cancer (SBBC) (273).

FOXP3 has been investigated as a direct therapeutic target in preclinical and early clinical studies. Intratumoral administration of an adenoviral vector encoding P60 (AdP60) in breast-tumor-bearing mice significantly reduced Treg infiltration, delayed tumor growth, and inhibited spontaneous lung metastases, suggesting that this gene-therapy-mediated blockade of FOXP3 could enhance the response of these tumors to standard treatments (116). Scientists have found that histone deacetylase (HDAC) inhibitors upregulated the expression of FOXP3 and increased the number and function of FOXP3+ CD4+ CD25+ Treg (274). Moreover, in a TNBC model, HDAC inhibitors upregulated PD-L1 and human leukocyte antigen DR (HLA-DR) on tumor cells and down-regulated CD4+ FOXP3+ Treg, which resulted in a significant decrease in tumor growth and increased survival (275). Furthermore, pharmacology and biological effects of drug named AZD8701 was tested in both cultured Tregs and mouse syngeneic tumor model, taking a big step toward clinical treatment. By one or two-way analysis of variance with multiple comparisons, statistical significance of biological effects was evaluated and scientists discovered that AZD8701 led to a dose-dependent knockdown of FOXP3 in primary Tregs, reduction of suppressive immune function and efficient target downregulation in humanized mice at clinically relevant doses, indicating that antisense inhibitors such as AZD8701 might be a promising candidate for further clinical trials. According to the reported essay, AZD8701 is now being evaluated clinically as a first-in-class FOXP3 inhibitor for the treatment of cancer in phase 1 clinical trial (NCT04504669), where monotherapy of AZD8701 and a combination therapy of AZD8701 plus durvalumab were evaluated (276). Other than immunotherapies, scientists also discovered that a dual clustered regularly interspaced short palindromic repeats (CRISPR) interference and activation targeting to X inactive specific transcript and FOXP3 loci altered their transcription and their nearby epigenetic modifications, offering a potential therapeutic for female breast cancer (277).

As extensive *in vitro* research in this field underscores the importance of FOXP3 as a promising biomarker for breast cancer, several drugs targeting breast cancer are advancing toward clinical or preclinical trials. However, through all these experiments, due to the complex mechanisms of FOXP3 toward cancer, the number of drugs targeting FOXP3 is limited and more clinical research are needed. Although the majority of research indicate that FOXP3 might be an important factor for poor prognosis, further studies are needed to clarify mechanisms behind controversial results (278).

### 3.3 Drugs targeting WT1

WT1-peptide vaccine and WT1-targeted DC vaccine represent the principal forms of WT1-immunotherapy, with their efficacy supported by multiple studies. A retrospective study including 313 patients with advanced cancer has confirmed the broad efficacy of WT1 peptide therapy for a wide range of advanced cancers (279). Based on phase I data, an article confirms that the potential toxicities of weekly WT1-immunotherapy are acceptable and indicates a potential anti-tumor effect on solid malignancy (280). Therefore, WT1-immunothrapy might be a potential candidate for future clinical immunology on solid cancer (281, 282).

Research has been conducted to explore the relationship between WT1 expression levels and tumor aggressiveness, providing evidence and theories for WT1-immunotherapy. In invasive breast cancer with >90% mucinous component, WT1 expression was found to be significantly related with low-intermediate nuclear/histological grade, ER positivity, HER2 negativity and a lower Ki-67 proliferation (283). There were no significant differences in WT1 expression in myoepithelial cells in normal breast tissue and breast cancer cells (284). In a case-control study with 959 female subjects, the hypermethylation of WT1 has been shown to be associated with an increased risk of luminal subtypes of breast cancer (285). Studies have been conducted to assess the impact of WT1-immunotherapy on cancer regression. WT1 vaccination was reported to be capable of inducing WT1-specific cytotoxic T lymphocytes without damage to normal tissues in patients with several kinds of cancer including breast cancer, though the number of patients was relatively small and in need of further research to verify the conclusion (286). In an *in vitro* study, scientists demonstrated for the first time that WT1 peptide vaccination plus Mycobacterium bovis bacillus Calmette-Guérin cell wall skeleton (BCG-CWS) was more effective in eradicating WT1-expressing tumor cells in mice model, suggesting this combination may be applicable to cancer immunotherapy in clinical settings (287).

In addition to *in vitro* studies, clinical trials are advancing in the field of WT1-immunotherapy. In a clinical trial which involved ten patients with advanced cancer, participants received a WT1 peptide-pulsed DC vaccination biweekly with toxicity, and clinical and immunological responses serving as the endpoints. All AEs were tolerable under an adjuvant setting and tumor shrinkage was observed in three patients, providing initial clinical evidence that this vaccination is potentially effective for patients with advanced cancer (288). Additionally, a novel clinical study evaluated the efficacy of WT1 immunotherapy. In a randomized phase I study (NCT01220128), WT1-immunotherapy was used together with standard therapy in patients with WT1-positive Stage II/III breast cancer as neoadjuvant treatment. In this trial, patients were divided into 4 groups to be treated differently. Patients who were post-menopausal and HR-positive were allocated to group A to receive aromatase inhibitor, while group B was composed of patients receiving chemotherapy. Group D was for HR-positive/HER2-negative patients on chemotherapy, whereas group C included HER2-overexpressing patients on trastuzumab-chemotherapy combination. Different teams were treated differently on the use of WT1-immunotherapeutic. Group A-C were randomized in a 2:1 ratio to be treated with the combination of 6 or 8 doses of WT1-immunotherapeutic and standard neoadjuvant treatment in a double-blind manner. However, group D received WT1-immunotherapeutic in an open-label manner. Focusing on the evaluation of safety and immunogenicity, the study found that in group where patients were co-treated with neoadjuvant therapy, the combination of WT1-immunotherapeutic and standard therapy was well tolerated and led to WT1-specfic antibody response. In contrast, humoral response in patients receiving NACT or trastuzumab together with chemotherapy was impaired, suggesting that WT1-immnuotherapeutic had its best effect in certain occasions (289).

While several studies have confirmed the efficacy of WT1-immunotherapy in advanced cancers including breast cancer, the evidence remains preliminary due to the limited patient numbers in each trial and the recency of the treatment. Further research involving larger and more diverse patient cohorts is warranted.

## 4 Toxicity and safety of neoadjuvant immune checkpoint inhibitors in breast cancer

The integration of immune checkpoint inhibitors (ICIs) into neoadjuvant chemotherapy regimens for triple-negative breast cancer (TNBC) has improved pathologic complete response rates, yet this therapeutic advance is accompanied by substantial immune-related toxicity. In the curative-intent setting, where long-term quality of life and treatment completion are paramount, characterization of agent-specific adverse event profiles, real-world toxicity burdens, and evidence-based management strategies is essential.

### 4.1 Comparative toxicity profiles across checkpoint inhibitors

Four phase 3 trials define the current safety landscape of neoadjuvant ICIs in breast cancer: KEYNOTE-522 (pembrolizumab), IMpassion031 (atezolizumab), GeparNuevo (durvalumab), and CheckMate 7FL (nivolumab). KEYNOTE-522 reported grade 3–5 immune-related adverse events (irAEs) in 13.0% of pembrolizumab-treated patients versus 1.0% in the chemotherapy-alone arm (181, 224, 225). Residual irAEs persisted in approximately 19% of patients at last assessment, with 14% requiring long-term thyroid hormone replacement. IMpassion031 demonstrated a more favorable safety profile: grade 3–4 adverse events were balanced between arms (63% vs. 60%), no treatment-related deaths were reported, and discontinuation rates were comparable (12.8% vs. 11.4%) (173). GeparNuevo exhibited a 47% rate of any-grade thyroid dysfunction among durvalumab-treated patients, the highest reported in neoadjuvant breast cancer trials (178).

CheckMate 7FL revealed a distinct toxicity profile: serious adverse events were nearly doubled with nivolumab (22.9% vs. 12.9%), and two fatal irAEs—pneumonitis and hepatitis—occurred 51–61 days after the final dose. Rare but severe events including Guillain–Barré syndrome (0.4%) and myocarditis (0.4%) were also observed, requiring vigilant post-treatment surveillance and a low threshold for emergent neurologic or cardiac evaluation (179). A network meta-analysis of randomized trials in neoadjuvant TNBC confirmed agent-specific differences in safety profiles, with pembrolizumab carrying the highest risk of grade ≥3 adverse events and atezolizumab appearing relatively better tolerated (290).

### 4.2 Toxicity and treatment discontinuation

Real-world evidence demonstrates that toxicity-driven treatment modifications occur more frequently in clinical practice than in trial cohorts. In KEYNOTE-522, 13.4% of patients discontinued pembrolizumab due to adverse events. By contrast, a multicenter real-world study of 153 patients across four US academic institutions reported a 21% pembrolizumab discontinuation rate and approximately 50% of patients received reduced relative dose intensity (RDI) of chemotherapy, with 54% of patients experiencing any irAE and 12% grade ≥3 events (291). A French single-center cohort of 92 patients documented 29.3% immunotherapy discontinuation due to irAEs, with grade 3–4 events including hepatitis (8.6%), colitis (3.3%), myocarditis (2%), and autoimmune hemolytic anemia (1%) (292). An ambispective study of 366 patients at a comprehensive cancer center reported dose reductions in 48.7% versus 25.0% with chemotherapy alone, treatment delays in 65.2% versus 27.5%, and permanent discontinuation in 31.1% versus 16.0% (293).

Lower relative dose intensity (RDI) of chemotherapy was independently associated with reduced pathologic complete response rates (odds ratio 1.53 for full vs. reduced RDI, P = 0.03), suggesting that preserving chemotherapy intensity is more important for achieving pathologic response than preserving immunotherapy cycles (291). Delayed irAEs affected 25% of patients, with 7.5% occurring after treatment discontinuation—These events occurred within the 90-day period but nevertheless emphasize the importance of sustained post-treatment surveillance (294). The delayed fatal events in CheckMate 7FL (pneumonitis and hepatitis at 51–61 days post-final dose) exemplify this risk and support extended vigilance beyond the standard 90-day window, particularly for nivolumab-treated patients.

### 4.3 Organ-specific toxicities and management

Thyroid dysfunction is the most common irAE across neoadjuvant breast cancer trials, occurring in 15–47% of patients depending on the agent and monitoring intensity (178, 224, 295). Hypothyroidism is generally manageable with levothyroxine replacement and rarely requires treatment discontinuation (296); however, in the curative-intent setting, lifelong hormone replacement in up to 15% of patients constitutes a meaningful long-term burden (225).

Hepatotoxicity is of particular concern when ICIs are combined with CDK4/6 inhibitors or anthracyclines. CheckMate 7A8, evaluating nivolumab plus palbociclib and anastrozole in ER-positive/HER2-negative breast cancer, was closed prematurely due to 28.6% grade 3/4 hepatic adverse events and 42.9% treatment discontinuation, demonstrating unacceptable synergistic toxicity that precludes further investigation of this combination (257). Pneumonitis, though uncommon (reported in 2% of patients receiving neoadjuvant pembrolizumab for breast cancer and approximately 3.6% in patients with non-small cell lung cancer receiving PD-1 inhibitors), is potentially fatal and accounts for a substantial proportion of ICI-related deaths (297, 298). The delayed fatal case in CheckMate 7FL underscores that new respiratory symptoms during or after therapy should prompt immediate chest CT evaluation and high-dose corticosteroid therapy. Dermatologic toxicity, primarily maculopapular rash and pruritus, occurred in 32.6% of patients in a real-world cohort and in approximately 22% of patients in KEYNOTE-522 (224, 292). Gastrointestinal toxicity, including colitis and diarrhea, is more prevalent in real-world practice than in trial reports and may require infliximab for refractory cases (292). Severe endocrinopathies—particularly adrenal insufficiency and hypopituitarism—occurred in approximately 3% of KEYNOTE-522 patients and can be life-threatening if unrecognized; education on symptoms of adrenal crisis before treatment initiation is essential.

Management of irAEs follows the ASCO 2021 guideline framework: grade 1 permits continuation of the checkpoint inhibitor with close monitoring; grade 2 requires holding therapy and initiating corticosteroids (prednisone 0.5–1 mg/kg/day); grade 3 mandates high-dose steroids (1–2 mg/kg/day) with a minimum 4–6 week taper; and grade 4 necessitates permanent discontinuation, with the exception of endocrinopathies controlled by hormone replacement (296). In the neoadjuvant setting, early intervention is critical because reactive dose reductions or delays in chemotherapy may compromise pathologic response.

### 4.4 Clinical recommendations

The integration of immune checkpoint inhibitors into neoadjuvant chemotherapy necessitates a systematic approach to toxicity surveillance, early pharmacologic intervention, and longitudinal follow-up to mitigate treatment-related morbidity.Baseline and serial laboratory monitoring should include thyroid function assessment every 4–6 weeks and hepatic enzyme evaluation before each chemotherapy cycle (296). Early corticosteroid intervention at grade 2 irAEs (prednisone 0.5–1 mg/kg/day or equivalent) has been demonstrated to prevent progression to higher-grade toxicities and to preserve chemotherapy dose intensity, thereby protecting pathologic complete response rates; this approach is reinforced by consensus recommendations from both ASCO and SITC (299). For patients manifesting complex or multi-organ toxicities, multidisciplinary collaboration with endocrinology, pulmonology, dermatology, and gastroenterology remains integral to comprehensive irAE management, reflecting the multidisciplinary care models endorsed by ESMO and ASCO (300). Preemptive patient education regarding the clinical manifestations of irAEs prior to treatment initiation facilitates earlier recognition and reporting, thereby shortening the time to diagnostic evaluation and therapeutic intervention. Furthermore, sustained post-treatment surveillance for delayed irAEs is warranted, particularly following nivolumab exposure, given the documented occurrence of fatal pneumonitis and hepatitis at 51–61 days post-final dose in CheckMate 7FL and the 7.5% incidence of post-treatment irAEs observed in real-world breast cancer cohorts (179, 294). Implementation of these monitoring and management strategies serves to optimize the therapeutic index of neoadjuvant immunotherapy while minimizing the risk of persistent or permanent organ dysfunction in patients treated with curative intent.

## 5 Other emerging biomarkers in neoadjuvant immunotherapy

Beyond PD-L1, several biomarkers are reshaping patient selection and response monitoring in neoadjuvant TNBC immunotherapy. Tumor mutational burden (TMB) measures somatic mutations per megabase, higher TMB generates more neoantigens and may predict better response to PD-1 blockade (301, 302). Tumor-infiltrating lymphocytes, particularly CD8+ cytotoxic T cells, reflect pre-existing anti-tumor immunity. Higher TIL density correlates with better prognosis after neoadjuvant immunochemotherapy (303). Circulating tumor DNA (ctDNA) allows non-invasive longitudinal monitoring of residual disease and early detection of resistance mutations. Prospective trials in early breast cancer have shown that ctDNA dynamics during neoadjuvant therapy can stratify relapse risk and identify patients unlikely to achieve pathological complete response (304, 305). Microsatellite instability (MSI) and mismatch repair deficiency identify hypermutated phenotypes sensitive to PD-1 blockade, though their prevalence in breast cancer is lower than in colorectal or endometrial cancer, limiting broad applicability despite the tissue-agnostic approval of pembrolizumab (306, 307).

Acquired resistance to PD-1/PD-L1 monotherapy has aroused interest in co-inhibitory receptors and myeloid checkpoints that sustain immunosuppression. LAG-3, frequently co-expressed with PD-1 on exhausted CD8+ T cells, limits early T-cell activation and has been independently associated with poor response to checkpoint inhibitors (308, 309). TIGIT and TIM-3 constitute additional exhaustion axes: TIGIT restrains both T-cell and NK-cell effector function by competing with CD226 for CD155 (310), whereas TIM-3 marks terminally differentiated exhausted T cells and dampens antitumor immunity through galectin-9 engagement (311). At the myeloid level, TREM2-expressing tumor-associated macrophages establish an immunosuppressive niche that physically obstructs T-cell entry and suppresses cytotoxic function (312, 313). How these biomarkers should be optimally combined into prospective trial designs remains an active area of investigation.

## 6 Limitations and future perspectives

### 6.1 Limitations

Findings from various preclinical and clinical studies have encouraged the application of neoadjuvant immunotherapy in breast cancer. However, challenges and limitations still remain regarding the outcomes of the clinical studies conducted thus far (172, 314). Although drugs targeting PD-1/PD-L1 have been intensively evaluated, such as KEYNOTE-522 and IMpassion031 where more than 300 patients were included, neoadjuvant strategies targeting FOXP3 or WT1 remain underexplored (174, 197). Investigators leaned toward identifying FOXP3 as a predictive biomarker more than a targeting one, leading to the lack of clinical trials toward breast cancer (315, 316). Additionally, WT1 peptide vaccines lack regulatory approval for breast cancer, constraining prospective trial design and patient access.

Regarding the inconsistent findings from trials studying atezolizumab as we described above, a standardized study design is recommended, which addresses different endpoints, number of participants and statistical methods to enhance the reliability of the results. For instance, as an important factor in data analysis, different endpoints may even shift the conclusion to the opposite side. Long-term effects of drugs were not evaluated in some experiments, thus possible AEs could be ignored. Differences between districts and patient-reported outcomes (PROs) were less studied, making it inconvenient to treat patients separately in the real world (317, 318).

Besides, Biomarker-based patient selection remains a central unresolved challenge. Intratumoral heterogeneity of PD-L1 expression and TIL density can produce discordant results between core biopsy and surgical specimens, limiting single-lesion assessment (319, 320). PD-L1 status is also dynamic: neoadjuvant chemotherapy reshapes both PD-L1 levels and immune infiltrates from baseline to residual disease, so a single pretreatment measurement may not reflect the biology that determines NACT response (321). Compounding this, commercially available PD-L1 immunohistochemistry assays use different antibody clones, scoring algorithms, and positivity thresholds, and concordance across platforms—especially for immune cell staining in breast cancer—remains imperfect (322, 323).

Long-term survival data from phase 3 neoadjuvant trials remain immature, and whether pCR reliably predicts durable cure across all breast cancer subtypes is not established. In the curative-intent setting, immune-related adverse events represent a clinically relevant liability: delayed toxicities can emerge several months to years after treatment completion, yet robust tools to prospectively identify patients at high risk for severe irAEs are lacking (296). Beyond the clinic, the high cost of checkpoint inhibitors widens the gap in global access. In low- and middle-income countries, where the burden of triple-negative breast cancer is disproportionately heavy, affordability remains a decisive barrier to implementation (324, 325).

Though conclusions and results of each clinical study are clear, the underlying cause for the effectiveness or failure of the drugs used, or the interaction between new and existed therapies are mostly unknown. Further exploration of the mechanisms would facilitate our understanding of the results, resolve disagreement among different trials, and more importantly, refine therapies to achieve better effect.

### 6.2 Future perspectives

Drugs targeting PD-1/PD-L1 demonstrate promising therapeutic efficacy as neoadjuvant therapy in breast cancer, particularly in patients with TNBC who typically have poor prognosis due to high rates of systemic recurrence after conventional therapy, lacking adjuvant or neoadjuvant treatment options (326–329). A roadmap for future neoadjuvant approaches in HR+/HER2- breast cancer was also proposed, giving more evidence for clinical treatment (330–332). Thus, therapies targeting immunosuppressive molecules which are widely expressed on tumor cells such as PD-1 and PD-L1 are among the most promising strategies to reshape the TME, enabling immune cells to more efficiently kill cancer cells (333, 334).

Although several immunotherapies have proven effective in various malignancies, many patients do not respond to such treatments or eventually develop resistance, leading to unfavorable prognosis (319, 335, 336). In order to achieve better therapeutic effects, combination therapy has emerged and has become a trend in current cancer immunotherapy. Initial results from some experiments, such as KEYNOTE-522 and NeoTRIP-aPDL1 trials, demonstrated that chemotherapy might influence the interaction and treatment effect of immune checkpoint inhibitors on early-stage TNBC, providing rationale for the clinical application of such combinations (337, 338). Window-of-opportunity trials with serial biopsies enable rapid pharmacodynamic readouts and early responder identification without delaying standard therapy (339). Standardizing PD-L1 assay platforms and cutoff definitions would facilitate regulatory harmonization. To overcome primary resistance, dual checkpoint blockade—such as combined LAG-3 and PD-1 inhibition, which improved PFS in melanoma (340)—hold promise for application in breast cancer treatment. Similarly, TIGIT inhibition combined with PD-L1 blockade has shown early promise (341). Oncolytic viruses, tumor-infiltrating lymphocyte therapy, and CAR-T cells are also under early-phase evaluation (342–344).

Furthermore, recent progress in the evaluation methods largely improves the evaluation efficiency of neoadjuvant immunotherapies. When investigating new drugs, a neoadjuvant setting is preferred because drug efficacy can be evaluated earlier using pCR as an alternative endpoint for survival. The frequency and means of the treatment can be adjusted as soon as possible. This evaluation strategy is based on the observed results that pCR after neoadjuvant therapy significantly correlates with PFS and OS, which are the most important endpoints to evaluate the prognosis of patients with tumor (345).

Technological advances are refining tumor-immune microenvironment mapping. Spatial transcriptomics and multiplexed ion beam imaging have demonstrated that immune cell topography and compartmentalization predict immunotherapy response in breast cancer (319, 346). Integrating these spatial data with single-cell transcriptomics and machine learning-based computational pathology may yield multi-omic classifiers that surpass individual biomarkers (347, 348). Patient-derived organoids preserve tumor heterogeneity and can be co-cultured with autologous immune cells to enable personalized ex vivo screening of biomarker-drug pairings (349, 350), potentially reducing the requirement for large phase 3 trials. Collectively, these strategies may narrow the translational gap between evidence and clinical practice.

## 7 Conclusion

In conclusion, neoadjuvant immunotherapy is a promising treatment strategy for breast cancer patients and a good combination partner for traditional anti-cancer treatments. Amongst numerous immunotherapeutic targets evaluated in breast cancer, PD-L1/PD-1 inhibitors have been intensively studied and proved effective in a number of trials focusing on breast cancer patients. However, due to factors such as the difference of endpoints, the inadequate number of participants, or varying treatment regimen, studies may yield contradictory conclusions on effectiveness and toxicity of evaluated neoadjuvant immunotherapies. Thus, a standardized for study design is recommended for future studies (282).

## Glossary

ADCC: Antibody-dependent cellular cytotoxicity
AKT: Protein kinase B
APC: Antigen-presenting cell
ASCO: American Society of Clinical Oncology
ATAD3A-PINK1: ATPase family AAA domain-containing protein 3–PTEN-induced putative kinase 1
BATF: Basic leucine zipper transcriptional factor ATF-like
CAR-T: Chimeric antigen receptor T cells
CDK: Cyclin-dependent kinase
CKAP2L: Cytoskeleton-associated protein 2-like
COX-2: Cyclooxygenase-2
CRISPR: Clustered regularly interspaced short palindromic repeats
CSN5: COP9 signalosome complex subunit 5
CTL: Cytotoxic T lymphocyte
ctDNA: Circulating tumor DNA
DC: Dendritic cell
DDFS: Distant disease-free survival
DLT: Dose-limiting toxicity
EMT: Epithelial-to-mesenchymal transition
EGFR: Epidermal growth factor receptor
ERK: Extracellular-signal-regulated kinase
ESMO: European Society for Medical Oncology
FDA: Food and Drug Administration
FOXP3: Forkhead box protein P3
H3K4me3: Tri-methylation of histone H3 on lysine 4
HDAC: Histone deacetylase
HER2+: Human epidermal growth factor receptor 2-positive
HLA-DR: Human leukocyte antigen DR
HR+: Hormone receptor-positive
HUVEC: Human umbilical vein endothelial cell
HUWE1: HECT, UBA and WWE domain-containing protein 1
ICI: Immune checkpoint inhibitor
IDD EPC: Intensive dose-dense epirubicin, paclitaxel, and cyclophosphamide
IGF-IR: Insulin-like growth factor I receptor
iDFS: Invasive disease-free survival
IFN-γ: Interferon gamma
irAE: Immune-related adverse event
ITSM: Immunoreceptor tyrosine-based switch motif
ITT: Intention-to-treat
JAK: Janus kinase
KAT2B: Lysine acetyltransferase 2B
KGF: Keratinocyte growth factor
LAG-3: Lymphocyte-activation gene 3
LAPTM5: Lysosomal protein transmembrane 5
m6A: N6-methyladenosine
MEK: MAP kinase-ERK kinase
METTL3: Methyltransferase 3, N6-adenosine-methyltransferase complex catalytic subunit
MLL1: Mixed-lineage leukemia 1
MSI: Microsatellite instability
mPGES1: Microsomal prostaglandin E synthase-1
mTOR: Mammalian target of rapamycin
MTA1: Metastasis-associated gene 1
NACT: Neoadjuvant chemotherapy
NF-AT: Nuclear factor of activated T-cells
NK: Natural killer
NSCLC: Non-small cell lung cancer
ORR: Objective response rate
OS: Overall survival
PARP: Poly-ADP-ribose polymerase
PBMC: Peripheral blood mononuclear cell
PD-1: Programmed cell death protein 1
PD-L1: Programmed cell death ligand 1
PFS: Progression-free survival
PGE2: Prostaglandin E2
PGRN: Progranulin
PI3K: Phosphatidylinositol 3-kinase
PIS: Primary immunologic score
PLD: Pegylated liposomal doxorubicin
PRO: Patient-reported outcome
pCR: Pathological complete response
PTEN: Phosphatase and tensin homolog
RBMS3: RNA binding motif single stranded interacting protein 3
RDI: Relative dose intensity
Runx1: Runt-related transcription factor 1
SHP-2: SH2-containing protein tyrosine phosphatase 2
SITC: Society for Immunotherapy of Cancer
STAT3: Signal transducer and activator of transcription 3
STIM1: Stromal interaction molecule 1
STT3A: STT3 oligosaccharyltransferase complex catalytic subunit A
T-VEC: Talimogene laherparepvec
TAM: Tumor-associated macrophage
TBM-1: Tubeimoside-1
TCR: T cell receptor
TGF-β: Transforming growth factor-β
TIGIT: T cell immunoreceptor with Ig and ITIM domains
TIM-3: T cell immunoglobulin and mucin-domain containing-3
TIL: Tumor-infiltrating lymphocyte
TMB: Tumor mutational burden
TME: Tumor microenvironment
TMUB1: Transmembrane and ubiquitin-like domain-containing protein 1
TNBC: Triple-negative breast cancer
Treg: Regulatory T cell
TREM2: Triggering receptor expressed on myeloid cells 2
VISTA: V-domain Ig-containing suppressor of T-cell activation
WT1: Wilms’
Wnt: Wingless/Int-1
YAP: Yes-associated protein
ZAP70: Zinc finger antiviral protein 70
ZEB2: Zinc finger E-box binding homeobox 2
Zbtb7a: Zinc finger and BTB domain containing 7A gene
c-SMAC: Central supramolecular activation cluster.
