# Supplementary material for: Biomarker-driven neoadjuvant immunotherapy in triple-negative breast cancer: emerging therapeutic targets FOXP3 and WT1
Source: Front Immunol. 2026 Aug 10;17:1851289. doi: 10.3389/fimmu.2026.1851289 (PMC13501238; doi:10.3389/fimmu.2026.1851289)
Supplement: Supplementary file 1 [file Table1.docx]

| **Table 2. Ongoing clinical studies assessing the efficacy of neoadjuvant immunotherapy in breast cancer** | | | | | | | | | | | |
| --- | --- | --- | --- | --- | --- | --- | --- | --- | --- | --- | --- |
| **Identifier** | **Trial name** | **Phase** | **Country** | **Sample size** | **Molecular Subtype** | **Biomarker Status** | **Trial design** | **Primary endpoint** | **Key Outcomes** | **Major Adverse Events** | **Estimated Completion Date** |
| NCT03281954 | - | Ⅲ | United States | 1550 | TNBC | All-comers | Randomized, Parallel Assignment, Triple-blind | EFS | Study status: active, not recruiting (SABCS 2024: 4-yr EFS 85.2% vs 81.9%, P=0.08) | Not yet fully reported (SABCS 2024 preliminary) | 2027/11/30 |
|  |  |  |  |  |  |  | Arm A:  Placebo |  |  |  |  |
|  |  |  |  |  |  |  | Arm B:  Atezolizumab |  |  |  |  |
| NCT03756298 | - | Ⅱ | Korea | 284 | TNBC | All-comers | Randomized, Parallel Assignment, Triple-blind | 5-yr iIDFS rate | Study status: active, not recruiting (Primary completion: Dec 2026) | Not yet reported | 2027/1/31 |
|  |  |  |  |  |  |  | Arm A:  Atezolizumab + Capecitabine |  |  |  |  |
|  |  |  |  |  |  |  | Arm B:  Capecitabine alone |  |  |  |  |
| NCT05498896 | BARBICAN | Ⅱ | United Kingdom | 146 | TNBC | All-comers | Randomized, Parallel Assignment, Open-label | pCR | Study status: active, not recruiting (Primary completion: Aug 2026) | Not yet reported | 2026-01-30 |
|  |  |  |  |  |  |  | Arm A:  Atezolizumab + Chemotherapy |  |  |  |  |
|  |  |  |  |  |  |  | Arm B:  Atezolizumab + Chemotherapy + Ipatasertib |  |  |  |  |
| NCT05332561 | COGNITION-GUIDE | Ⅱ | Germany | 240 | High-risk early BC (residual disease) | Biomarker-selected (7-arm umbrella) | Non-Randomized, Parallel Assignment, Open-label | IDFS in the Entire Study Population Four Years after Surgery | Study status: recruiting (Primary completion: Mar 2030) | Not yet reported | 2030/12/1 |
|  |  |  |  |  |  |  | Arm 1:  Atezolizumab (Immune Evasion) |  |  |  |  |
|  |  |  |  |  |  |  | Arm 2:  Inavolisib (PI3K) |  |  |  |  |
|  |  |  |  |  |  |  | Arm 3:  Ipatasertib (AKT) |  |  |  |  |
|  |  |  |  |  |  |  | Arm 4:  Olaparib (PARP, DNA-Repair) |  |  |  |  |
|  |  |  |  |  |  |  | Arm 5:  Sacituzumab Govitecan (TROP-2) |  |  |  |  |
|  |  |  |  |  |  |  | Arm 6:  Trastuzumab/Pertuzumab (ERBBB) |  |  |  |  |
|  |  |  |  |  |  |  | Arm 7:  Observation |  |  |  |  |
| NCT06175390 | SKYLINE | Ⅱ | France | 81 | TNBC (early + metastatic) | All-comers | Non-Randomized, Parallel Assignment, Open-label | Incidence of Treatment-Emergent Adverse Events | Study status: recruiting (Primary completion: Aug 2026) | Not yet reported | 2029/2/15 |
|  |  |  |  |  |  |  | Cohort A:  early Triple Negative Breast Cancer | In cohort A, Rate of pCR |  |  |  |
|  |  |  |  |  |  |  | Cohort B:  metastatic Triple Negative Breast Cancer | In cohort B, Progression-free survival rate (6mPFS) |  |  |  |
| NCT04849364 | PERSEVERE | Ⅱ | United States | 197 | TNBC (residual disease) | ctDNA-directed | Non-Randomized, Parallel Assignment, Open-label | 2 Year DFS of Arm 1 | Study status: active, not recruiting (Primary completion: Sep 2027) | Not yet reported | 2034/1/31 |
|  |  |  |  |  |  |  | Arm 1a:  Patients who are ctDNA-positive and harbor a genomic target. DNA repair pathway = talazoparib + capecitabine (CLOSED) |  |  |  |  |
|  |  |  |  |  |  |  | Arm 1b:  Patients who are ctDNA-positive and harbor a genomic target. Immunotherapy pathway = pembrolizumab + capecitabine (CLOSED) |  |  |  |  |
|  |  |  |  |  |  |  | Arm 1c:  Patients who are ctDNA-positive and harbor a genomic target. PI3K Pathway = inavolisib + capecitabine ---> +/- standard of care pembrolizumab |  |  |  |  |
|  |  |  |  |  |  |  | Arm 1d:  Patients who are ctDNA-positive and harbor a genomic target. DNA Repair + Immunotherapy = talazoparib + capecitabine +/- standard of care pembrolizumab |  |  |  |  |
|  |  |  |  |  |  |  | Arm 2:  Subjects have plasma ctDNA positive but do not have a genomically driven treatment option |  |  |  |  |
|  |  |  |  |  |  |  | Arm 3:  Subjects have plasma ctDNA negative |  |  |  |  |
| NCT05629585 | TROPION-Breast03 | Ⅲ | United States, Brazil, China, Canada and so on | 1174 | TNBC (non-pCR) | All-comers | Randomized, Parallel Assignment, Open-label | iDFS for Dato-DXd + Durvalumab vs. ICT |  |  |  |
|  |  |  |  |  |  |  | Arm 1:  Dato-DXd in combination with Durvalumab |  | Study status: recruiting (Primary completion: Nov 2028) | Not yet reported | 2030/3/27 |
|  |  |  |  |  |  |  | Arm 2:  Dato-DXd |  |  |  |  |
|  |  |  |  |  |  |  | Arm 3:  Investigators Choice Therapy |  |  |  |  |
| NCT06112379 | - | Ⅲ | Uniated States, Austria, Brazil and so on | 1800 | TNBC / HR-low/HER2- | All-comers | Randomized, Parallel Assignment, Open-label | pCR and EFS in the Experimental vs Control Arms | Study status: recruiting (Primary completion: Nov 2028) | Not yet reported | 2030/8/28 |
|  |  |  |  |  |  |  | Experimental:  Dato-DXd plus durvalumab |  |  |  |  |
|  |  |  |  |  |  |  | Active Comparator:  Pembrolizumab plus chemotherapy |  |  |  |  |
| NCT05633654 | ASCENT-05/AFT-65 OptimICE-RD/NSABP B-63 | Ⅲ | United States | 1514 | TNBC (residual disease) | All-comers | Randomized, Parallel Assignment, Open-label | iDFS | Study status: recruiting (Primary completion: 2028) | Not yet reported | 2031/8/1 |
|  |  |  |  |  |  |  | Experimental:  Sacituzumab govitecan-hziy (SG) + Pembrolizumab |  |  |  |  |
|  |  |  |  |  |  |  | Active Comparator:  Treatment of Physician's Choice (TPC): Pembrolizumab or Pembrolizumab + Capecitabine |  |  |  |  |
| NCT06358573 | INVINCIBLE-4-SAKK | Ⅱ | Switzerland | 61 | TNBC | All-comers | Randomized, Parallel Assignment, Open-label | pCR Using the Definition of ypT0/Tis ypN0 | Study status: recruiting (SABCS 2024/2025: preliminary pCR 71.4% vs 33%) | Preliminary: 44% fewer Grade ≥3 AEs in Cohort A vs B | 2029/12/1 |
|  |  |  |  |  |  |  | Cohort A:  2 Injections of INT230-6 into primary tumor of the breast+neoadjuvant immuno-chemotherapy |  |  |  |  |
|  |  |  |  |  |  |  | Cohort B:  neoadjuvant immuno-chemotherapy |  |  |  |  |
| NCT01042379 | I-SPY | Ⅱ | United States | 5000 | All subtypes | Biomarker-selected | Randomized, Parallel Assignment, Open-label | Determine whether adding experimental agents to standard neoadjuvant medications increases the probability of pCR over standard neoadjuvant chemotherapy for each biomarker signature established at trial entry | Study status: recruiting (Platform trial; multiple arms published) | Varies by arm; pembrolizumab arm: manageable safety profile | 2031/12/1 |
|  |  |  |  |  |  |  | Experimental:  Experimental agents + Standard neoadjuvant |  |  |  |  |
|  |  |  |  |  |  |  | Active Comparator:  Standard Therapy |  |  |  |  |
| NCT03740893 | PHOENIX | Ⅱ | United Kingdom | 81 | All subtypes (umbrella) | Biomarker-selected (cohort-specific) | Randomized, Parallel Assignment, Open-label | Cohorts B and C co-primary endpoint 1: Change in Mean Proliferation Index | Study status: Ongoing (Extended) — Recruiting (2025-08 update) | Not yet reported | 2025/12/1  (Extended) |
|  |  |  |  |  |  |  | No Intervention:  Cohort A (standard care reference cohort) | Cohorts B and C co-primary endpoint 2: Changes in the Proliferation Gene Expression Signature |  |  |  |
|  |  |  |  |  |  |  | Experimental:  Cohort B  (AZD6738 monotherapy) | Cohort D co-primary endpoint 1: Change in CD8+ TILs |  |  |  |
|  |  |  |  |  |  |  | Experimental:  Cohort C  (olaparib monotherapy) | Cohort D co-primary endpoint 2: Changes in the IFNγ+ Signature |  |  |  |
|  |  |  |  |  |  |  | Experimental:  Cohort D  (durvalumab monotherapy) |  |  |  |  |
| NCT05498155 | OlympiaN | Ⅱ | Uniated States, Australia, Belgium and so on | 50 | HER2-negative BRCAm | BRCA-mutated | Non-Randomized, Parallel Assignment, Open-label | pCR Rate | Study status: active, not recruiting (Primary completion: Nov 2024; SABCS 2025: pCR 68% vs 80%) | Not yet reported | 2026/12/31 |
|  |  |  |  |  |  |  | Cohort A:  Neoadjuvant Olaparib monotherapy group |  |  |  |  |
|  |  |  |  |  |  |  | Cohort B:  Neoadjuvant combination therapy with olaparib plus durvalumab |  |  |  |  |
| NCT05215106 | POP-Durva | Ⅱ | France | 200 | TNBC (small, early-stage) | All-comers | Single Group Assignment, Open Label | pCR rate after 2 Administrations of Durvalumab | Study status: recruiting (Primary completion: 2026-06) | Not yet reported | 2026/6/1 |
|  |  |  |  |  |  |  | Experimental:  Durvalumab |  |  |  |  |
| NCT03875573 | Neo-CheckRay | Ⅱ | France， Belgium | 147 | Luminal B (MammaPrint high-risk) | All-comers (MammaPrint-selected) | Randomized, Parallel Assignment, Open-label | Evaluation of the Immune Related or Radiation Therapy related Toxicity of Special Interest | Study status: Recruiting (Safety run-in published JITC 2023; ESMO 2024: RCB 0-1 primary endpoint not met) | Safety run-in: manageable (JITC 2023) | 2029/9/30 |
|  |  |  |  |  |  |  | Arm 1:  Chemotherapy and radiotherapy | Evaluation of the Feasibility of the Primary Surgery |  |  |  |
|  |  |  |  |  |  |  | Arm 2:  Chemotherapy and pre-operative radiotherapy plus durvalumab | Demonstration of the Tumor Response in Arms 2 or 3 versus Arm 1 |  |  |  |
|  |  |  |  |  |  |  | Arm 3:  Chemotherapy & pre-op radiotherapy + durvalumab + oleclumab |  |  |  |  |
| NCT04331067 | - | I/Ⅱ | United States | 50 | TNBC | All-comers | Randomized, Parallel Assignment, Open-label | Percent Change in TILs | Study status: Active, not recruiting (Arm B cabiralizumab closed; Amended to single-arm nivolumab + chemo) | Not yet reported (cabiralizumab arm discontinued Oct 2022) | 2026/5/31 |
|  |  |  |  |  |  |  | Active Comparator:  Arm A:  Neoadjuvant chemo + nivolumab | Percent Change in TAMs |  |  |  |
|  |  |  |  |  |  |  | Experimental:  Arm B:  Neoadjuvant chemo + nivolumab + cabiralizumab | Safety of the Regimen as Measured by Incidence of Adverse Events (Safety lead-in Only) |  |  |  |
|  |  |  |  |  |  |  | Experimental:  Unrandomized Arm: Neoadjuvant Chemo + Nivolumab |  |  |  |  |
| NCT03546686 | - | II | Uniated States | 80 | TNBC | All-comers | Single Group Assignment, Open-label | EFS | Study status: Ongoing (Extended) — Recruiting; results not yet published | Not yet reported | 2028/6/1 |
|  |  |  |  |  |  |  | Arm 1:  Pembrolizumab + Core Biopsy/Cryoablation + Breast Surgery +Post Surgery pembrolizumab or Ipilimumab and Nivolumab + Core Biopsy/Cryoablation + Breast Surgery +Post Surgery Nivolumab |  |  |  |  |
| NCT05681728 | - | Ⅱ | China | 50 | TNBC | All-comers | Single Group Assignment, Open-label | pCR rate using the Definition of ypT0/Tis ypN0 | Study status: recruiting (Primary completion: 2026-08) |  | 2024/6/1  (Extended) |
|  |  |  |  |  |  |  | Experimental:  Pembrolizumab combined with chemotherapy |  |  |  |  |
| NCT06402435 | - | Ⅱ | - | 50 | Luminal-type | All-comers | Single Group Assignment, Open-label | pCR rate | Study status: Recruiting (Primary completion: 2026-08) | Not yet reported | 2027/9/1 |
|  |  |  |  |  |  |  | Experimental:  luminal-type breast cancer patients with Lvonescimab |  |  |  |  |
| NCT06418139 | PEMCLOCK | Observational | - | Not specified | TNBC | All-comers | Observational, Cross-Sectional | RCB | Study status: not yet recruiting (Primary completion: 2026-09) | Not applicable | 2026/9/1 |
| NCT05877859 | - | Observational | United States | 10 | TNBC | All-comers | Observational, prospective | Change in the Cytokine Milieu in the Blood | Study status: recruiting (Primary completion: 2027-92) | Not applicable | 2026/4/10 |
| NCT06448169 | - | Observational | - | 200 | TNBC | All-comers | Observational, prospective | pCR rate | Study status: not yet recruiting (Primary completion: Feb 2026) | Not applicable | 2026/4/30 |
| NCT01513408 | PRIMUNEO | Observational | France | 500 | All subtypes | All-comers (CD8/Foxp3 biomarker study) | Single group assignment, Open-label | OS | Study status: active, not recruiting; Primary completion 2015-10; multiple publications from cohort | Not applicable | 2025/10/1  (Extended) |
|  |  |  |  |  |  |  | Experimental:  CD8/Foxp3 |  |  |  |  |
| NCT05645380 | NeoTRACT | Ⅱ | United States | 100 | TNBC | sTIL-high (≥30%) / sTIL-intermediate / sTIL-low | Non-Randomized, Parallel Assignment, Open-label, Phase II | pCR rate in High sTIL Cohort with Radiographic Complete Response | Study status: recruiting; results not yet published (Original completion: 2024-11) | Not yet reported | 2024/12/1  (Extended) |
|  |  |  |  |  |  |  | Active Comparator:  High sTILs (≥30%) |  |  |  |  |
|  |  |  |  |  |  |  | Active Comparator:  Intermediate sTILs (5-29%) |  |  |  |  |
|  |  |  |  |  |  |  | Active Comparator:  Low sTILs (<5%) |  |  |  |  |
| NCT03515798 | PELICAN | Ⅱ | France | 52 | TNBC (inflammatory) | All-comers | Randomized, Parallel Assignment, Open-label, Phase II | Central evaluation of pCR rate | Study status: active, not recruiting (Primary completion: Aug 2022) | Not yet fully reported (Preliminary data published in PMC 2024) | 2027/8/22  (Estimated) |
|  |  |  |  |  |  |  | Experimental:  Pembrolizumab | Dose Limiting Toxicity (DLT) rates |  |  |  |
|  |  |  |  |  |  |  | Active Comparator:  Standard neoadjuvant chemotherapy |  |  |  |  |
| NCT06318897 | - | Ⅱ | United States | 28 | TNBC (Stage I cT1b-T1cN0M0) | All-comers | Randomized, Single Group Assignment, Open-label, Phase II | Safety and AEs | Study status: recruiting (Primary completion: 2027-09) | Not yet reported | 2025/9/30  (Estimated) |
|  |  |  |  |  |  |  | Experimental:  Pathologic Complete Response |  |  |  |  |
|  |  |  |  |  |  |  | Experimental:  Non-Pathologic Complete Response |  |  |  |  |
