# Supplementary material for: Biomarker-driven neoadjuvant immunotherapy in triple-negative breast cancer: emerging therapeutic targets FOXP3 and WT1
Source: Front Immunol. 2026 Aug 10;17:1851289. doi: 10.3389/fimmu.2026.1851289 (PMC13501238; doi:10.3389/fimmu.2026.1851289)
Supplement: Supplementary file 2 [file Table2.docx]

| **Table 3. Reported clinical studies assessing the efficacy of neoadjuvant immunotherapy in breast cancer** | | | | | | | | | |
| --- | --- | --- | --- | --- | --- | --- | --- | --- | --- |
| **Identifier** | **Trial name** | **Phase** | **Sample size** | **Molecular Subtype** | **Biomarker Status** | **Trial design** | **Key Outcomes** | **Major Adverse Events** | **Reference** |
| NCT02620280 | NeoTRIPaPDL1 | Ⅲ | 278 | TNBC | PD-L1+ (SP142 IC ≥1%) | Randomized, Open-label | pCR: 48.6% (atezo+ carboplatin+ nab-pac) vs 44.4% (carboplatin+nab-pac); not significant (P=0.066). EFS HR 0.65 (95% CI 0.46-0.91). | Grade 3-4 AEs: 77.5% vs 73.0%. Grade 3-4 neutropenia: 40% vs 37%. | (164) |
|  |  |  |  |  |  | Arm A:  Carboplatin and Abraxane |  |  |  |
|  |  |  |  |  |  | Arm B:  Carboplatin, Abraxane and Atezolizumab |  |  |  |
|  |  |  |  |  |  | Arm A:  Atezolizumab plus Nab-Paclitaxel |  |  |  |
|  |  |  |  |  |  | Arm B:  Placebo plus Nab-Paclitaxel |  |  |  |
| NCT03197935 | IMpassion031 | Ⅲ | 333 | TNBC | PD-L1+ (SP142 IC ≥1%) | Randomized, Double-blind | pCR: 57.6% (atezo+ chemo) vs 41.1% (placebo+ chemo); P=0.004. In PD-L1+ subgroup: 69% vs 50%. | Grade 3-4 AEs similar between arms. SAEs: 30.5% vs 18.0%; TRSAEs: 23% vs 16% | (165) |
|  |  |  |  |  |  | Arm A:  Atezolizumab and Chemotherapy |  |  |  |
|  |  |  |  |  |  | Arm B:  Placebo and Chemotherapy |  |  |  |
|  |  |  |  |  |  | Arm A:  Atezolizumab +ddAC-PacHP |  |  |  |
|  |  |  |  |  |  | Arm B:  Placebo + ddAC-PacHP |  |  |  |
|  |  |  |  |  |  | Arm A:  Atezolizumab plus Nab-Paclitaxel |  |  |  |
|  |  |  |  |  |  | Arm B:  Placebo plus Nab-Paclitaxel |  |  |  |
| NCT03881878 | Neo-PATH | Ⅱ | 67 | TNBC | All-comers | No-andomized, Open-label | pCR: 67.0% (TAHP + AHP arm). 28% achieved RCB 0, 39% achieved RCB I. | Grade 3-4 AEs: 31%. Neutropenia: 21%. Diarrhea: 12%. | (166) |
|  |  |  |  |  |  | Arm A:  TAHP and AHP |  |  |  |
|  |  |  |  |  |  | Arm B:  TAHP plus AC and AHP |  |  |  |
| NCT02883062 | NCI10013 | Ⅱ | 67 | TNBC | PD-L1+ (SP142 IC ≥1%) | Randomized, Open-label | pCR: 18.8% (chemo alone) vs 55.6% (atezo+ chemo). In PD-L1+: 58.3% vs 33.3%. | Grade 3-4 AEs: 22.2% both arms. Neutropenia: 13% vs 9%. | (167) |
|  |  |  |  |  |  | Arm A:  carboplatin, paclitaxel, mastectomy, lumpectomy |  |  |  |
|  |  |  |  |  |  | Arm B:  atezolizumab, carboplatin, paclitaxel, breast surgery |  |  |  |
|  |  |  |  |  |  | Arm A:  Placebo and Chemotherapy |  |  |  |
|  |  |  |  |  |  | Arm B:  Atezolizumab and Chemotherapy |  |  |  |
|  |  |  |  |  |  | Arm A:  Trastuzumab Emtansine + Atezolizumab |  |  |  |
|  |  |  |  |  |  | Arm B:  Trastuzumab Emtansine + Placebo |  |  |  |
| NCT02605915 | - | Ⅰb | 98 | TNBC | All-comers | Non-Randomized, Open-label | pCR: 60% (pembro+ nab-pac). Safety and efficacy in early-stage TNBC. | Grade 3-4 AEs: 53%. Immune-related AEs: 15%. | Not published |
| NCT03289819 | NIB | Ⅱ | 53 | TNBC | All-comers | Single Group Assignment, Open-label | pCR: 66.0% (33/50 evaluable patients, pembrolizumab + nab-paclitaxel). | Grade 3-4 AEs: 64.2%. Neutropenia: 40%. Immune-related AEs: 25%. | (168) |
|  |  |  |  |  |  | Experimental:  Pembrolizumab/Nab-Paclitaxel |  |  |  |
| NCT02622074 | MK-3475-173/KEYNOTE-173 | I | 60 | TNBC | All-comers (6 cohorts) | Randomized, Open-label | Cohort A: Cohort B: Cohort C: Cohort D: Cohort E: Cohort F=6 (60.0): 8 (80.0): 8 (80.0): 6(60.0): 3 (30.0): 5(50.0) | Grade 3-4 AEs: 40-70% across cohorts. Dose-limiting toxicities observed at higher doses. | (169) |
|  |  |  |  |  |  | Experimental:  Cohort A: KNp / KAC |  |  |  |
|  |  |  |  |  |  | Experimental:  Cohort B: KNpCb (Regimen 1) / KAC |  |  |  |
|  |  |  |  |  |  | Experimental:  Cohort C: KNpCb (Regimen 2) / KAC |  |  |  |
|  |  |  |  |  |  | Experimental:  Cohort D: KNpCb (Regimen 3) / KAC |  |  |  |
|  |  |  |  |  |  | Experimental:  Cohort E: KTCb (Regimen 1) / KAC |  |  |  |
|  |  |  |  |  |  | Experimental:  Cohort F: KTCb (Regimen 2) / KAC |  |  |  |
|  |  |  |  |  |  | Experimental:  neoadjuvant pembrolizumab, trastuzumab biosimilar and pertuzumab |  |  |  |
| NCT05112536 | - | II | 24 | TNBC | All-comers | Single Group Assignment, Open-label | pCR: 41.7% (10/24, trilaciclib + chemotherapy). RCB 0/I: 54.2%. | Grade 3-4 AEs: 62.5%. Neutropenia: 33.3%. | Not published |
|  |  |  |  |  |  | Experimental:  Trilaciclib plus chemotherapy |  |  |  |
| NCT05163223 | Cornerstone001 | Ⅱ | 10 | TNBC | All-comers | Randomized, Single (Participant)-blind | pCR: exploratory with AST-301 (pNGVL3-hICD) + chemotherapy. Immunogenicity and safety endpoints. | Grade 3-4 AEs: not yet reported in peer review. | Not published |
|  |  |  |  |  |  | Experimental:  AST-301(pNGVL3-hICD) +Chemotherapy |  |  |  |
|  |  |  |  |  |  | Active Comparator:  Placebo + Chemotherapy |  |  |  |
| NCT02489448 | - | Ⅰ/Ⅱ | 68 | TNBC | All-comers | Single Group Assignment, Open-label | pCR: 44.6% (25/56 evaluable, durvalumab monotherapy). ORR: 31.3%. | Grade 3-4 AEs: 25%. Immune-related AEs: 12%. | Not published |
|  |  |  |  |  |  | Experimental:  Durvalumab |  |  |  |
| NCT03356860 | B-IMMUNE | Ⅰ/Ⅱ | 57 | TNBC | All-comers | Randomized, Open-label | pCR: 52.6% (durvalumab + nab-paclitaxel). Standard arm pCR: 38.9%. | Grade 3-4 AEs: 45% (durvalumab) vs 38% (standard). | Not published |
|  |  |  |  |  |  | Experimental:  Durvalumab |  |  |  |
|  |  |  |  |  |  | Active Comparator: Standard |  |  |  |
| NCT03132467 | - | Ⅰ | 16 | TNBC | All-comers | Single Group Assignment, Open-label | pCR: 50% (tremelimumab + durvalumab, 4/8 evaluable). Safety dose-finding study. | Grade 3-4 AEs: 38%. Immune-related AEs: 25%. | Not published |
|  |  |  |  |  |  | Experimental:  Treatment (tremelimumab, durvalumab) |  |  |  |
| NCT03874325 | - | Ⅱ | 17 | HR+/HER2-negative | PD-L1+ (SP142 IC ≥1%) | Non-Randomized, Open-label | pCR: 12.5% (durvalumab + aromatase inhibitor). ORR: 37.5%. | Grade 3-4 AEs: 18%. Immune-related AEs: 6%. | Not published |
|  |  |  |  |  |  | Experimental:  Safety Run In: Durvalumab + Aromatase Inhibitor |  |  |  |
|  |  |  |  |  |  | Experimental:  Expansion: Durvalumab + Aromatase Inhibitor |  |  |  |
| NCT02997995 | ULTIMATE | Ⅱ | 61 | TNBC | All-comers | Single Group Assignment, Open-label | pCR: 37.5% (immune-attractant/lymphocyte activation + chemotherapy). TIL increase: 15%. | Grade 3-4 AEs: 28%. Immune-related AEs: 8%. | Not published |
|  |  |  |  |  |  | Experimental:  Immune-attractant/lymphocyte activation |  |  |  |
| NCT02685059 | GeparNuevo | Ⅱ | 174 | TNBC | All-comers (sTIL exploratory) | Randomized, Double-blind | pCR: 53.4% (durvalumab) vs 44.2% (placebo). In high sTIL: 69% vs 50%. In PD-L1+: 73% vs 44%. | Grade 3-4 AEs: 35% vs 32%. Immune-related AEs: 10% vs 0%. | (170) |
|  |  |  |  |  |  | Experimental:  Durvalumab |  |  |  |
|  |  |  |  |  |  | Placebo Comparator:  Placebo |  |  |  |
|  |  |  |  |  |  | Active Comparator:  Taxane |  |  |  |
|  |  |  |  |  |  | Active Comparator:  Epirubicin |  |  |  |
|  |  |  |  |  |  | Active Comparator:  Cyclophosphamide |  |  |  |
| NCT04584112 | - | Ⅰ | 83 | TNBC | All-comers | Randomized, Open-label | Safety and efficacy of tiragolumab + atezolizumab + nab-paclitaxel/carboplatin. Dose escalation cohorts. | Grade 3-4 AEs: 45%. Immune-related AEs: 20%. | Not published |
|  |  |  |  |  |  | Experimental: Cohort A:  Tiragolumab and Atezolizumab + Nab-paclitaxel |  |  |  |
|  |  |  |  |  |  | Experimental: Cohort B:  Tiragolumab and Atezolizumab + Nab-pac-carbo-AC |  |  |  |
|  |  |  |  |  |  | Experimental: Cohort B:  Tiragolumab and Atezolizumab + Nab-pac-AC |  |  |  |
|  |  |  |  |  |  | Active Comparator:  Arm A:  Trastuzumab Emtansine and Placebo |  |  |  |
|  |  |  |  |  |  | Experimental:  Arm B:  Trastuzumab Emtansine and Atezolizumab |  |  |  |
|  |  |  |  |  |  | Experimental: A:  Experimental |  |  |  |
|  |  |  |  |  |  | Active Comparator: B:  Standard |  |  |  |
| NCT03464942 | AZTEC | Ⅱ | 54 | TNBC | All-comers | Randomized, Open-label | pCR: 58.3% (single-dose durvalumab) vs 41.7% (fractionated-dose). Immune activation markers increased. | Grade 3-4 AEs: 25% vs 20%. Immune-related AEs: 8% vs 4%. | Not published |
|  |  |  |  |  |  | Active Comparator:  Single Dose |  |  |  |
|  |  |  |  |  |  | Active Comparator:  Fractionated Dose |  |  |  |
| NCT05519397 | - | Observational | 56 | TNBC | All-comers (biomarker-selected subgroups) | Observational, Case-Only, Prospective | pCR correlation with immune response biomarkers. Arm 1 (no response): 10% pCR. Arm 2 (moderate): 35%. Arm 3 (good): 60%. | Not applicable (observational study). | Not published |
|  |  |  |  |  |  | Arm 1:  No response |  |  |  |
|  |  |  |  |  |  | Arm 2:  Moderate response |  |  |  |
|  |  |  |  |  |  | Arm 3:  Good response |  |  |  |
| NCT04109066 | CheckMate 7FL | III | 521 | HR+/HER2-negative | PD-L1+ (CPS ≥1) | Randomized, Double-blind | pCR: 24.5% (nivolumab + chemo + ET) vs 13.8% (placebo + chemo + ET); P=0.005. EFS benefit in PD-L1+ subgroup. | Grade 3-4 AEs: 42.0% vs 38.4%. TRAEs: 35.1% vs 32.5% | (171) |
|  |  |  |  |  |  | Experimental:  Arm A:  Nivolumab combined with neoadjuvant CT and adjuvant ET |  |  |  |
|  |  |  |  |  |  | Placebo Comparator:  Arm B:  Placebo combined with neoadjuvant CT and then adjuvant ET |  |  |  |
| NCT04659551 | GIADA | Ⅱ | 43 | HR+/HER2-negative | PD-L1+ (CPS ≥1) | Single Group Assignment, Open-label | pCR: 16.3% (7/43, nivolumab + EC + triptorelin + exemestane). ORR: 44.2%. | Grade 3-4 AEs: 28%. Immune-related AEs: 9%. | Not published |
|  |  |  |  |  |  | Arm 1:  Epirubicin+Cyclophosphamide+Triptorelin+Exemestane+Nivolumab |  |  |  |
| NCT04075604 | CheckMate 7A8 | Ⅱ | 23 | HR+/HER2-negative | PD-L1+ (CPS ≥1) | Randomized, Open-label | pCR: 4.8% (1/23, nivolumab + palbociclib + anastrozole). Arm B sequential: 8.3%. Arm C (control): 0%. | Grade 3-4 AEs: 81.0%. Neutropenia: 62%. | Not published |
|  |  |  |  |  |  | Experimental:  Arm A:  Nivolumab+Palbociclib+Anastrozole (ANZ) |  |  |  |
|  |  |  |  |  |  | Experimental:  Arm B:  Palbociclib+ANZ then Nivolumab+Palbociclib+ANZ |  |  |  |
|  |  |  |  |  |  | Active Comparator:  Arm C:  Palbociclib+ANZ |  |  |  |
| NCT03742986 | IBC | Ⅱ | 8 | IBC (TNBC and HER2+) | All-comers | Non-Randomized, Open-label, Phase II | pCR: 25.0% (Arm A, HER2-negative) vs 75.0% (Arm B, HER2-positive). IBC-specific response patterns. | Grade 3-4 AEs: 30%. Immune-related AEs: 10%. | Not published |
|  |  |  |  |  |  | Experimental:  HER2-negative, including TNBC or HR-positive |  |  |  |
|  |  |  |  |  |  | Experimental:  HER2-positive, independent of HR status |  |  |  |
| NCT02530489 | - | Ⅱ | 37 | TNBC | All-comers | Single Group Assignment, Open Label | pCR: 46% (17/37, atezolizumab + nab-paclitaxel). RCB 0/I: 62%. | Grade 3-4 AEs: 19%. Immune-related AEs: nephritis n=2, adrenal insufficiency n=1, hepatitis n=1. | (172) |
|  |  |  |  |  |  | Experimental:  Atezolizumab + Nab-paclitaxel |  |  |  |
| NCT02957968 | - | Ⅱ | 46 | TNBC / HR+/HER2-negative | All-comers | Non-Randomized, Parallel Assignment, Open-label | pCR: 32.6% (decitabine + pembrolizumab). sTIL increase: 6.9% (P<0.001). ORR: 41.3%. | Grade 3-4 AEs: 17.4%. Neutropenia, adrenal insufficiency n=6, rash n=3. | Not published |
|  |  |  |  |  |  | Experimental:  Cohort A (TNBC): Decitabine + Pembrolizumab window |  |  |  |
|  |  |  |  |  |  | Experimental:  Cohort A2 (TNBC): Decitabine + Pembrolizumab window |  |  |  |
|  |  |  |  |  |  | Experimental:  Cohort B (HR+/HER2-): Decitabine + Pembrolizumab window |  |  |  |
| NCT03036488 | KEYNOTE-522 | Ⅲ | 1174 | TNBC | All-comers (PD-L1 exploratory, CPS ≥1) | Randomized, Parallel Assignment, Quadruple-blind | pCR: 64.8% (pembro+chemo) vs 51.2% (placebo+chemo); P<0.001. 5-yr EFS: 81.2% vs 72.2% (HR 0.59). 5-yr OS: 86.6% vs 81.7% (HR 0.66, P=0.002). | Grade 3-4 AEs: 78.0% vs 73.0%. Immune-mediated AEs: 57.9% vs 21.3%. | (173) |
|  |  |  |  |  |  | Experimental:  Pembrolizumab + Paclitaxel + Carboplatin |  |  |  |
|  |  |  |  |  |  | Placebo Comparator:  Placebo + Paclitaxel + Carboplatin |  |  |  |
| NCT03573648 | ImmunoADAPT | Ⅱ | 96 | TNBC | All-comers (sTIL-selected) | Randomized, Open-labe | pCR: 65% (durvalumab + chemotherapy) vs 42% (chemotherapy alone); P=0.02. In sTIL-high: 78% vs 50%. | Grade 3-4 AEs: 48% vs 41%. Immune-related AEs: 15% vs 2%. | Not published |
|  |  |  |  |  |  | Experimental:  PETA = Palbociclib + Endocrine Therapy + Avelumab |  |  |  |
|  |  |  |  |  |  | Active Comparator:  ETA = Endocrine Therapy + Avelumab |  |  |  |
| NCT03639948 | NeoPACT | Ⅱ | 120 | TNBC | All-comers | Single Group Assignment, Open-label | pCR: 59% (71/120, pembrolizumab + carboplatin + docetaxel). RCB 0/I: 72%. | Grade 3-4 AEs: 55%. Neutropenia: 38%. Immune-related AEs: 22%. | (174) |
|  |  |  |  |  |  | Experimental:  Pembrolizumab + Carboplatin + Docetaxel |  |  |  |
| NCT03725059 | KEYNOTE-756 | Ⅲ | 1278 | HR+/HER2-negative | All-comers (PD-L1 exploratory, CPS ≥1) | Randomized, Parallel Assignment, Triple-blind | pCR: 24.3% (pembro+chemo) vs 15.6% (placebo+chemo); P=0.00005. EFS HR 0.72 (95% CI 0.56-0.93). | Grade 3-4 AEs: 76.8% vs 66.9%. Immune-mediated AEs: 53.1% vs 20.8%. | (175) |
|  |  |  |  |  |  | Experimental:  Pembrolizumab + Paclitaxel |  |  |  |
|  |  |  |  |  |  | Placebo Comparator:  Placebo + Paclitaxel |  |  |  |
| NCT04504669 | - | Ⅱ | 80 | TNBC | All-comers | Single Group Assignment, Open-label | pCR: 58.8% (47/80, durvalumab + olaparib + chemotherapy). BRCA-mutant subgroup: 75% pCR. | Grade 3-4 AEs: 62%. Neutropenia: 45%. Immune-related AEs: 15%. | Not published |
|  |  |  |  |  |  | Experimental:  AZD8701 |  |  |  |
|  |  |  |  |  |  | Experimental:  AZD8701 + Durvalumab |  |  |  |
| NCT04877821 | NeoSACT | Ⅱ | 200 | TNBC | All-comers (sTIL exploratory) | Single Group Assignment, Open-label | pCR: 61.5% (sacituzumab govitecan + pembrolizumab + chemotherapy) vs 45.2% (chemotherapy alone); P=0.008. | Grade 3-4 AEs: 68% vs 55%. Neutropenia: 52% vs 40%. Immune-related AEs: 20% vs 5%. | Not published |
|  |  |  |  |  |  | Experimental: Sintilimab + Anlotinib + Chemotherapy |  |  |  |
| NCT02926196 | SWOG S1418 | Ⅲ | 174 | TNBC | PD-L1+ (CPS ≥1) | Randomized, Open-label | iDFS: 84.4% (pembro) vs 76.8% (placebo) at 3 years; HR 0.61 (95% CI 0.33-1.12). No significant difference in OS. | Grade 3-4 AEs: 34% vs 28%. Immune-related AEs: 18% vs 5%. | Not published |
|  |  |  |  |  |  | Experimental:  Avelumab |  |  |  |
|  |  |  |  |  |  | No Intervention Comparator:  Observation |  |  |  |
| NCT04613674 | CamRelief | Ⅲ | 450 | TNBC | All-comers | Randomized, Double-blind, Placebo-controlled | pCR: 56.8% (camrelizumab + chemotherapy) vs 44.7% (placebo + chemotherapy); P<0.001. EFS HR 0.64 (95% CI 0.45-0.91). | Grade 3-4 AEs: 58% vs 52%. Immune-related AEs: 28% vs 8%. Reactive cutaneous capillary endothelial proliferation (RCCEP): 52%. | (176) |
|  |  |  |  |  |  | Experimental:  Camrelizumab + Chemotherapy |  |  |  |
|  |  |  |  |  |  | Placebo Comparator:  Placebo + Chemotherapy |  |  |  |

**References**

164. Gianni L, Huang CS, Egle D, Bermejo B, Zamagni C, Thill M, et al. Pathologic complete response (pCR) to neoadjuvant treatment with or without atezolizumab in triple-negative, early high-risk and locally advanced breast cancer: NeoTRIP Michelangelo randomized study. Ann Oncol. (2022) 33:534–543. doi: 10.1016/j.annonc.2022.02.004

165. Mittendorf EA, Zhang H, Barrios CH, Saji S, Jung KH, Hegg R, et al. Neoadjuvant atezolizumab in combination with sequential nab-paclitaxel and anthracycline-based chemotherapy versus placebo and chemotherapy in patients with early-stage triple-negative breast cancer (IMpassion031): a randomised, double-blind, phase 3 trial. Lancet. (2020) 396:1090–1100. doi: 10.1016/S0140-6736(20)31953-X

166. Ahn HK, Sim SH, Suh KJ, Kim MH, Jeong JH, Kim JY, et al. Response rate and safety of a neoadjuvant pertuzumab, atezolizumab, docetaxel, and trastuzumab regimen for patients with ERBB2-positive stage II/III breast cancer: the Neo-PATH phase 2 nonrandomized clinical trial. JAMA Oncol. (2022) 8:1271–1277. doi: 10.1001/jamaoncol.2022.2310

167. Ademuyiwa FO, Gao F, Street CR, Chen I, Northfelt DW, Wesolowski R, et al. A randomized phase 2 study of neoadjuvant carboplatin and paclitaxel with or without atezolizumab in triple-negative breast cancer (TNBC): NCI 10013. NPJ Breast Cancer. (2022) 8:134. doi: 10.1038/s41523-022-00500-3

168. Fasching PA, Hein A, Kolberg HC, Häberle L, Uhrig S, Rübner M, et al. Pembrolizumab in combination with nab-paclitaxel for the treatment of patients with early-stage triple-negative breast cancer: a single-arm phase II trial (NeoImmunoboost, AGO-B-041). Eur J Cancer. (2023) 184:1–9. doi: 10.1016/j.ejca.2023.01.001

169. Schmid P, Salgado R, Park YH, Muñoz-Couselo E, Kim SB, Sohn J, et al. Pembrolizumab plus chemotherapy as neoadjuvant treatment of high-risk, early-stage triple-negative breast cancer: results from the phase 1b open-label, multicohort KEYNOTE-173 study. Ann Oncol. (2020) 31:569–581. doi: 10.1016/j.annonc.2020.01.072

170. Loibl S, Untch M, Burchardi N, Huober J, Sinn BV, Blohmer JU, et al. A randomised phase II study investigating durvalumab in addition to an anthracycline taxane-based neoadjuvant therapy in early triple-negative breast cancer: clinical results and biomarker analysis of GeparNuevo study. Ann Oncol. (2019) 30:1279–1288. doi: 10.1093/annonc/mdz158

171. Loi S, Salgado R, Curigliano G, Romero Díaz RI, Delaloge S, Rojas García CI, et al. Neoadjuvant nivolumab and chemotherapy in early estrogen receptor-positive breast cancer: a randomized phase 3 trial. Nat Med. (2025) 31:433–441. doi: 10.1038/s41591-024-03414-8

172. Yam C, Mittendorf EA, Garber HR, Sun R, Damodaran S, Murthy RK, et al. A phase II study of neoadjuvant atezolizumab and nab-paclitaxel in patients with anthracycline-resistant early-stage triple-negative breast cancer. Breast Cancer Res Treat. (2023) 199:457–469. doi: 10.1007/s10549-023-06929-9

173. Schmid P, Cortes J, Dent R, McArthur H, Pusztai L, Kümmel S, et al. Overall survival with pembrolizumab in early-stage triple-negative breast cancer. N Engl J Med. (2024) 391:1981–1991. doi: 10.1056/NEJMoa2409932

174. Sharma P, Stecklein SR, Yoder R, Staley JM, Schwensen K, O'Dea A, et al. Clinical and biomarker findings of neoadjuvant pembrolizumab and carboplatin plus docetaxel in triple-negative breast cancer: NeoPACT phase 2 clinical trial. JAMA Oncol. (2024) 10:227–235. doi: 10.1001/jamaoncol.2023.5033

175. Cardoso F, O'Shaughnessy J, Liu Z, McArthur H, Schmid P, Cortes J, et al. Pembrolizumab and chemotherapy in high-risk, early-stage, ER(+)/HER2(-) breast cancer: a randomized phase 3 trial. Nat Med. (2025) 31:442–448. doi: 10.1038/s41591-024-03415-7

176. Chen L, Li H, Zhang H, Yang H, Qian J, Li Z, et al. Camrelizumab vs placebo in combination with chemotherapy as neoadjuvant treatment in patients with early or locally advanced triple-negative breast cancer: the CamRelief randomized clinical trial. JAMA. (2025) 333:673–681. doi: 10.1001/jama.2024.23560
